# Supplementary material for: Long-term outcomes of antenatal corticosteroids for preterm birth: An overview of systematic reviews
Source: PLOS Glob Public Health. 2025 May 7;5(5):e0004575. doi: 10.1371/journal.pgph.0004575 (PMC12057917; doi:10.1371/journal.pgph.0004575)
Supplement: S1 Table — (DOCX) [file pgph.0004575.s001.docx]

**S1 Table. AMSTAR-2 ratings of the included reviews**

| **Review ID** | **1** | **2** | **3** | **4** | **5** | **6** | **7** | **8** | **9** | **10** | **11** | **12** | **13** | **14** | **15** | **16** | **Overall rating** |
| --- | --- | --- | --- | --- | --- | --- | --- | --- | --- | --- | --- | --- | --- | --- | --- | --- | --- |
| McGoldrick 2020 | Y | Y | Y | Y | Y | Y | Y | Y | Y | Y | Y | Y | Y | Y | Y | Y | High |
| Sarid 2022 | Y | Y | N | Y | Y | Y | N | Y | Y | N | NA | NA | N | Y | NA | Y | Critically low |
| Ninan 2022 | Y | Y | N | Y | Y | Y | Y | Y | Y | N | Y | Y | Y | N | Y | Y | Moderate |
| Walters 2022 | Y | Y | Y | Y | Y | Y | Y | Y | Y | Y | Y | Y | Y | Y | Y | Y | High |
| Ciapponi 2021 | Y | Y | N | Y | Y | Y | Y | Y | Y | Y | Y | Y | Y | Y | Y | Y | High |
| Blankenship 2020 | Y | Y | N | Y | Y | Y | N | Y | Y | N | NA | NA | N | Y | NA | Y | Low |
| Crowther 2019 | Y | Y | Y | Y | Y | Y | Y | Y | Y | N | Y | Y | Y | Y | Y | Y | High |
| Park 2016 | Y | PY | N | Y | Y | Y | N | Y | Y | Y | Y | Y | Y | N | Y | Y | Low |
| Amiya 2016 | Y | PY | N | Y | Y | Y | N | Y | Y | N | Y | Y | Y | Y | Y | Y | Low |
| Sotiriadis 2015 | Y | Y | N | PY | N | Y | Y | Y | Y | N | Y | Y | Y | Y | Y | Y | Moderate |
| Peltoniemi 2011 | N | N | N | PY | Y | Y | N | N | N | N | Y | N | N | N | N | Y | Critically low |
| Onland 2011 | Y | N | N | PY | Y | Y | PY | N | Y | N | NA | NA | Y | Y | NA | N | Critically low |
| Aghajafari 2001 | Y | N | Y | PY | Y | Y | N | N | N | N | NA | NA | Y | Y | N | N | Critically low |
| Ninan 2023a | Y | Y | N | Y | Y | Y | Y | Y | Y | N | NA | NA | Y | Y | NA | Y | Moderate |
| Ninan 2023b | Y | Y | N | Y | Y | Y | Y | Y | Y | N | NA | NA | Y | Y | NA | Y | Moderate |
| Williams 2022 | Y | Y | Y | Y | Y | Y | Y | Y | Y | Y | Y | Y | Y | Y | Y | Y | High |
| Wang 2022 | Y | Y | N | PY | Y | Y | N | PY | Y | N | Y | Y | Y | Y | Y | Y | Low |
| Sacco 2022 | Y | PY | N | Y | Y | Y | N | N | Y | N | NA | NA | N | N | NA | Y | Critically low |
| CROWLEY 1995 | Y | N | Y | PY | N | N | N | N | N | N | Y | N | N | N | Y | N | Critically low |
