# Supplementary material for: Long-term outcomes of antenatal corticosteroids for preterm birth: An overview of systematic reviews
Source: PLOS Glob Public Health. 2025 May 7;5(5):e0004575. doi: 10.1371/journal.pgph.0004575 (PMC12057917; doi:10.1371/journal.pgph.0004575)
Supplement: S2 Table — (DOCX) [file pgph.0004575.s002.docx]

**S2 Table. Summary of reported outcomes in the included reviews**

| **Review ID** | **Outcome** | | | | | |
| --- | --- | --- | --- | --- | --- | --- |
|  | **Neurodevelopmental** | **Psychological** | **Physical growth** | **Respiratory** | **Cardiovascular** | **Survival/ mortality** |
| **Single/unspecified course of ACS vs. placebo/no treatment** | | | | | | |
| McGoldrick 2020 (1)  Ninan 2022 (2)  Park 2016 (3)  Sotiriadis 2015 (4)  Onland 2011 (5)  Crowley 1995 (6) | - Developmental delay (1, 3) - Neurodevelopmental impairment (2, 3) - Cerebral palsy (1, 2, 4) - Moderate/serious CP (2, 3) - Intellectual impairment (1) - BSID Mental Development Index-II <70 (2) - BSID III cognitive score <85 (2) - Hemiparesis (2) - Diparesis (2) - Moderate to severe functional disability (3) - Intact survival (4, 5) - Severe disability (4) - Minor disability (4) - Mental developmental index <70 (3, 4) - Psychomotor development index <70 (3, 4) - Bayley III cognitive score <70 (3) - Cognitive development –Wechsler Scales of Intelligence: Verbal, Performance, and Full Intelligence Quotient (4) - Hearing impairment (1, 2) - Visual impairment (1, 2) - Blindness (3) - Deafness (3) - Neurologic abnormality (6) | - Behavioural and learning difficulty (1) | - Body weight (1, 2) - Height (1, 2) - Head circumference (1, 2) | - Chronic lung disease at 2 years (3) - Allergic diseases (2) - Allergic rhinitis (2) - Asthma (2) | - Systolic blood pressure (1) | - Death in childhood (1) - Infant mortality at 18-22 months (3) |
| **Any ACS exposure (single/multiple/repeat courses) vs. placebo/no ACS** | | | | | | |
| Ninan 2023a (7)  Ninan 2022 (2)  Sacco 2022 (8)  Sarid 2022 (9)  Wang 2022 (10)  Blankenship 2020 (11)  Amiya 2016 (12) | - Speech/language development disorder (7) - Psychological developmental disorder (specified/unspecified) (7) - Epilepsy (7) - Motor function development disorder (7) - Scholastic skill development disorder (7) - Vision or hearing loss (7) - Developmental delay (7) - Neurodevelopmental impairment (2) - Suspected neurocognitive disorder (2) - Neurocognitive and neuromotor development (9) - Hearing impairment(2, 10) - Visual impairment(2) - Brain imaging(9) - Cerebral palsy(2, 7, 12) - Severe global delay up to three years of age(11) - General Development Quotient at 1 years' and 3 years’ follow up(12) - Survival without handicap(12) | - Any mental or behavioural disorder (ICD-10 codes F00-99)(2) - Psychiatric problems (9) - Abnormal behaviour (11, 12) - Attention deficit disorder (7) - Pervasive developmental disorder (7) | - Head circumference (9) - Growth <10th percentile in early childhood (12) - Weight >90^th^ & <10th percentile (7) - Height >90^th^ & <10^Th^ percentile (7) - BMI >90^th^ & <10^th^ percentile (7) | - Asthma (7) - Wheeze (7) - Hypoxemia (7) - Tachypnoea (7) - Bronchiolitis (7) - Albuterol use (7) | - Blood pressure (8) - Echocardiography (8) - Heart rate (8) - ECG (8) | - Survival at school age (12) |
| **Repeat/multiple courses of ACS vs. single course of ACS** | | | | | | |
| Ninan 2023b (13)  Walters 2022 (14)  Crowther 2019 (15)  Peltoniemi 2011 (16)  Aghajafari 2001 (17) | - Neurosensory disability (13) - Neurocognitive or neurobehavioral disability (13) - Neuromotor disability (13) - Developmental delay (13) - Neurodevelopmental impairment at early childhood follow up (14) - Neurocognitive impairment at mid- to later childhood follow up (14) - Developmental delay or intellectual impairment at early childhood follow-up (14, 15) - Cerebral palsy (13-17) - Survival free of neurodevelopmental impairment at early childhood follow up (14) - Survival free of neurodevelopmental impairment at early childhood follow up (14) - Survival free of major neurodevelopmental impairment at early childhood follow up (14) - Survival free of neurocognitive impairment at mid- to later childhood follow up (14) - Survival free of major neurocognitive impairment at mid- to later childhood follow up (14) - Gross motor dysfunction (15) - Death or neurosensory disability (15, 16) - Motor delay (15) - Any neurosensory disability (15) - Major neurosensory disability (15) - Cognitive impairment at mid- to later childhood follow-up(14) - Psychomotor Developmental Index at early childhood follow-up (14) - Mental Developmental Index at early childhood follow-up (14) - Deafness/hearing impairment (at early childhood follow-up and at mid- to later childhood follow-up) (14) - Blindness/visual impairment (at early childhood follow-up and at mid- to later childhood follow-up) (14) - Deafness (15) - Blindness (15) - Educational achievement at age five to less than 18 years (14) - Disability (17) | - Child behaviour at early childhood follow-up, Child Behaviour Checklist total score in the clinical range (14) - Child behaviour: Behaviour rating scale in the clinical range (BSID-II) (14) - Child behaviour at early childhood follow-up as assessed by Early Child Behaviour Questionnaire Extraversion summary scale (14) - Child behaviour at early childhood follow-up assessed by Early Child Behaviour Questionnaire Negative affectivity summary scale (14) - Child behaviour at early childhood follow-up assessed by Early Child Behaviour Questionnaire Effortful control summary scale (14) - Abnormal child behaviour at mid- to later childhood follow-up (14) - Child behaviour at mid- to later childhood follow-up (standardised mean difference) (14) - Child behaviour (15) | - Mean weight (13, 14, 16) - Mean weight z-score (14, 15) - Mean weight adjusted for age at early childhood follow‐up (standardised mean difference) (14) - Weight small for age at early childhood follow-up (14) - Mean head circumference (13, 14, 16) - Head circumference Z score (at early childhood follow-up and at mid- to later childhood follow-up) (14) - Head circumference small for age at early childhood follow-up (14) - Mean height (13, 14, 16) - Height Z score (14, 15) - Mean height adjusted for age at early childhood follow‐up (standardised mean difference) (14) - Height small for age at early childhood follow-up (14) - BMI Z scores at mid- to later childhood follow-up (14) - Body composition: total body fat-free mass at mid- to later childhood follow-up (14) - Body composition: total body fat mass at mid- to later childhood follow-up(14) - Weight (17) - Height (17) - Head circumference (17) | - Asthma or recurrent wheeze (14) - Respiratory disease (14, 15) - Measures of lung function at mid- to later childhood follow-up: mean FEV1 Z score (14) - Measures of lung function at mid- to later childhood follow-up: mean FVC Z score (14) - Measures of lung function at mid- to later childhood follow-up: mean FEV1/FVC Z score (14) | - Hypertension at early childhood follow-up (14) - Mean systolic blood pressure (14, 15) - Mean systolic blood pressure Z score (at early childhood follow-up and at mid- to later childhood follow-up) (14) - Mean diastolic blood (14, 15) - Mean diastolic blood pressure Z score (at early childhood follow-up and at mid- to later childhood follow-up) (14) - Follow up blood pressure (mean arterial) (15) | - Death (14, 15) |
| **ACS vs. ACS** | | | | | | |
| Williams 2022 (18)  Ciapponi 2021 (19) | - Neurodevelopmental disability at follow-up (18, 19) - Developmental delay – motor (mild/ moderate/severe) (18) - Developmental delay – motor (moderate/ severe only) (18) - Developmental delay – cognitive or language (moderate/ severe only) (18) - Developmental delay – cognitive or language (mild/ moderate/severe) (18) - Cerebral palsy (18) - Educational achievement – cognitive score (mean, BSID-III) (18) - Educational achievement – language score (mean, BSID-III) (18) - Educational achievement – motor score (mean, BSID-III) (18) - Visual impairment (18) - Hearing impairment (18) | - Childhood behaviour checklist (mean total score) (18) | - Weight at 2 years (18) - Height at 2 years (18) - Head circumference at 2 years (18) |  | - Systolic blood pressure (18) - Diastolic blood pressure (18) | - Death in childhood (18) |

1. McGoldrick E, Stewart F, Parker R, Dalziel SR. Antenatal corticosteroids for accelerating fetal lung maturation for women at risk of preterm birth. Cochrane Database of Systematic Reviews. 2020(12).

2. Ninan K, Liyanage SK, Murphy KE, Asztalos EV, McDonald SD. Evaluation of Long-term Outcomes Associated With Preterm Exposure to Antenatal Corticosteroids: A Systematic Review and Meta-analysis. JAMA Pediatr. 2022;176(6):e220483.

3. Park CK, Isayama T, McDonald SD. Antenatal Corticosteroid Therapy Before 24 Weeks of Gestation: A Systematic Review and Meta-analysis. Obstet Gynecol. 2016;127(4):715-25.

4. Sotiriadis A, Tsiami A, Papatheodorou S, Baschat AA, Sarafidis K, Makrydimas G. Neurodevelopmental Outcome After a Single Course of Antenatal Steroids in Children Born Preterm: A Systematic Review and Meta-analysis. Obstet Gynecol. 2015;125(6):1385-96.

5. Onland W, de Laat MW, Mol BW, Offringa M. Effects of antenatal corticosteroids given prior to 26 weeks' gestation: a systematic review of randomized controlled trials. Am J Perinatol. 2011;28(1):33-44.

6. Crowley PA. Antenatal corticosteroid therapy: a meta-analysis of the randomized trials, 1972 to 1994. Am J Obstet Gynecol. 1995;173(1):322-35.

7. Kiran N, Anja G, Yanchen W, Elizabeth VA, Marc B, Kellie EM, et al. The proportions of term or late preterm births after exposure to early antenatal corticosteroids, and outcomes: systematic review and meta-analysis of 1.6 million infants. BMJ. 2023;382:e076035.

8. Sacco A, Cornish EF, Marlow N, David AL, Giussani DA. The effect of antenatal corticosteroid use on offspring cardiovascular function: A systematic review. Bjog. 2023;130(4):325-33.

9. Sarid EB, Stoopler ML, Morency A-M, Garfinkle J. Neurological implications of antenatal corticosteroids on late preterm and term infants: a scoping review. Pediatric Research. 2022;92(5):1225-39.

10. Wang Y, Tian Q, Huang Y. Is antenatal corticosteroids exposure associated with hearing loss in preterm infants? A systematic review and meta-analysis. Am J Otolaryngol. 2023;44(1):103548.

11. Blankenship SA, Brown KE, Simon LE, Stout MJ, Tuuli MG. Antenatal corticosteroids in preterm small-for-gestational age infants: a systematic review and meta-analysis. Am J Obstet Gynecol MFM. 2020;2(4):100215.

12. Amiya RM, Mlunde LB, Ota E, Swa T, Oladapo OT, Mori R. Antenatal Corticosteroids for Reducing Adverse Maternal and Child Outcomes in Special Populations of Women at Risk of Imminent Preterm Birth: A Systematic Review and Meta-Analysis. PLoS One. 2016;11(2):e0147604.

13. Ninan K, Liyanage SK, Murphy KE, Asztalos EV, McDonald SD. Long-Term Outcomes of Multiple versus a Single Course of Antenatal Steroids: A Systematic Review. Am J Perinatol. 2023(EFirst).

14. Walters A, McKinlay C, Middleton P, Harding JE, Crowther CA. Repeat doses of prenatal corticosteroids for women at risk of preterm birth for improving neonatal health outcomes. Cochrane Database Syst Rev. 2022;4(4):Cd003935.

15. Crowther CA, Middleton PF, Voysey M, Askie L, Zhang S, Martlow TK, et al. Effects of repeat prenatal corticosteroids given to women at risk of preterm birth: An individual participant data meta-analysis. PLoS Med. 2019;16(4):e1002771.

16. Peltoniemi OM, Kari MA, Hallman M. Repeated antenatal corticosteroid treatment: a systematic review and meta-analysis. Acta Obstet Gynecol Scand. 2011;90(7):719-27.

17. Aghajafari F, Murphy K, Willan A, Ohlsson A, Amankwah K, Matthews S, et al. Multiple courses of antenatal corticosteroids: a systematic review and meta-analysis. Am J Obstet Gynecol. 2001;185(5):1073-80.

18. Williams MJ, Ramson JA, Brownfoot FC. Different corticosteroids and regimens for accelerating fetal lung maturation for babies at risk of preterm birth. Cochrane Database of Systematic Reviews. 2022(8).

19. Ciapponi A, Klein K, Colaci D, Althabe F, Belizán JM, Deegan A, et al. Dexamethasone versus betamethasone for preterm birth: a systematic review and network meta-analysis. American Journal of Obstetrics & Gynecology MFM. 2021;3(3):100312.
