# Supplementary material for: Long-term outcomes of antenatal corticosteroids for preterm birth: An overview of systematic reviews
Source: PLOS Glob Public Health. 2025 May 7;5(5):e0004575. doi: 10.1371/journal.pgph.0004575 (PMC12057917; doi:10.1371/journal.pgph.0004575)
Supplement: S3 Table — (DOCX) [file pgph.0004575.s003.docx]

**S3 Table. Results of meta-analyses conducted by included systematic reviews**

| **Review ID** | **Outcome category** | **Outcome** | **Intervention** | **No. of studies and children included in analysis** | **Gestational age at birth** | **Timing of assessment/age at follow up** | **Pooled effect estimates** | **Certainty of evidence**  **(GRADE)** |
| --- | --- | --- | --- | --- | --- | --- | --- | --- |
| **Single course of ACS vs. placebo/no treatment** | | | | | | | | |
| McGoldrick 2020 | Neurodevelopmental | Developmental delay in childhood | Single course of ACS vs. placebo/no treatment | 3 studies, 600 children | Mean GA 24 to 36 weeks | 2 to 12 years | RR 0.51, 95% CI 0.27 to 0.97 | Moderate |
| McGoldrick 2020 | Neurodevelopmental | Intellectual impairment | Single course of ACS vs. placebo/no treatment | 3 studies, 778 children | Mean GA 24 to 36 weeks | 3 to 12 years | RR 0.86, 95% CI 0.44 to 1.69 |  |
| McGoldrick 2020 | Neurodevelopmental | Cerebral palsy in childhood | Single course of ACS vs. placebo/no treatment | 5 studies, 904 children | Mean GA 24 to 36 weeks | 2 to 12 years | RR 0.60, 95% CI 0.34 to 1.03 |  |
| McGoldrick 2020 | Neurodevelopmental | Hearing impairment | Single course of ACS vs. placebo/no treatment | 2 studies, 166 children | Mean GA 24 to 32 weeks | 4 to 12 years | RR 0.64, 95% CI 0.04 to 9.87 |  |
| McGoldrick 2020 | Neurodevelopmental | Visual impairment | Single course of ACS vs. placebo/no treatment | 2 studies, 166 children | Mean GA 24 to 32 weeks | 4 to 12 years | RR 0.55, 95% CI 0.24 to 1.23 |  |
| McGoldrick 2020 | Psychological | Behavioural and learning difficulty | Single course of ACS vs. placebo/no treatment | 1 study, 90 children | Mean GA 26 to 32 weeks | 10 to 12 years | RR 0.86, 95% CI 0.35 to 2.09 |  |
| McGoldrick 2020 | Physical growth | Mean childhood weight (kg) | Single course of ACS vs. placebo/no treatment | 2 studies, 333 children | Mean GA 24 to 36 weeks | 6 to 12 years | MD 0.30, 95% CI -0.39 to 1.00 |  |
| McGoldrick 2020 | Physical growth | Mean childhood head circumference (cm) | Single course of ACS vs. placebo/no treatment | 2 studies, 328 children | Mean GA 24 to 36 weeks | 6 to 12 years | MD 0.27, 95% CI -0.08 to 0.63 |  |
| McGoldrick 2020 | Physical growth | Mean childhood height (cm) | Single course of ACS vs. placebo/no treatment | 2 studies, 334 children | Mean GA 24 to 36 weeks | 6 to 12 years | MD 1.02, 95% CI -0.26 to 2.29 |  |
| McGoldrick 2020 | Cardiovascular | Mean childhood systolic blood pressure (mmHg) | Single course of ACS vs. placebo/no treatment | 1 study, 223 children | Mean 24 to 36 weeks | 6 years | MD -1.06, 95% CI -4.06 to 0.86 |  |
| McGoldrick 2020 | Survival/mortality | Death in childhood | Single course of ACS vs. placebo/no treatment | 4 studies, 1010 children | Mean GA 24 to 36 weeks | Up to 6 years | RR 0.68, 95% 0.36 to 1.27 |  |
| Ninan 2022 | Neurodevelopmental | Neurodevelopmental impairment | Single course of ACS vs no ACS | 2 studies, 3948 children | Preterm | 18 to 22 months corrected age | aOR 0.69, 95% CI 0.57 to 0.84 | Low |
| Ninan 2022 | Neurodevelopmental | Cerebral palsy | Single course of ACS vs. no ACS | 2 studies | Preterm | 18 to 22 months corrected age | aOR 0.60, 95% CI 0.43 to 0.83 | Low |
| Ninan 2022 | Neurodevelopmental | Moderate/serious CP | Single course of ACS vs. no ACS | 2 studies | Preterm | 18 to 22 months corrected age | aOR 0.82, 95% CI 0.56 to 1.19 | Low |
| Ninan 2022 | Neurodevelopmental | Auditory impairment | Single course of ACS vs. no ACS | 3 studies | Preterm | 12 to 24 months corrected age | aOR 0.58, 95% CI 0.33 to 1.01 | Very low |
| Ninan 2022 | Neurodevelopmental | Visual impairment | Single course of ACS vs. no ACS | 3 studies | Preterm | 12 to 24 months corrected age | aOR 1.42, 95% CI 0.57 to 3.54 | Very low |
| Ninan 2022 | Neurodevelopmental | Cerebral palsy (unadjusted) | Single course of ACS vs. no ACS | 4 studies, 4050 children | Preterm | 18 to 22 months corrected age/after birth | RR 0.73, 95% CI 0.56 to 0.96 | Low |
| Ninan 2022 | Neurodevelopmental | BSID MDI-II <70 (unadjusted) | Single course of ACS vs. no ACS | 4 studies, 1265 children | Preterm | 18 months to 5 years | RR 0.94, 95% CI 0.72 to 1.23 | Very low |
| Ninan 2022 | Neurodevelopmental | BSID III cognitive score <85 (unadjusted) | Single course of ACS vs. no ACS | 3 studies, 3039 children | Preterm | 18 months to 24 months corrected age | RR 0.56, 95% CI 0.28 to 1.13 | Very low |
| Ninan 2022 | Neurodevelopmental | Auditory impairment (unadjusted) | Single course of ACS vs. no ACS | 4 studies, 4177 children | Preterm | 18 months to 24 months corrected age | RR 0.63, 95% CI 0.37 to 1.07 | Very low |
| Ninan 2022 | Neurodevelopmental | Visual impairment (unadjusted) | Single course of ACS vs. no ACS | 4 studies, 4173 children | Preterm | 18 months to 24 months corrected age | RR 1.33, 95% CI 0.58 to 3.08 | Very low |
| Ninan 2022 | Neurodevelopmental | Hemiparesis (unadjusted) | Single course of ACS vs. no ACS | 2 studies, 965 children | Preterm | 12 months to 24 months corrected age | RR 0.83, 95% CI, 0.14 to 5.02 | Very low |
| Ninan 2022 | Neurodevelopmental | Diparesis (unadjusted) | Single course of ACS vs. no ACS | 2 studies, 965 children | Preterm | 12 months to 24 months corrected age | RR 0.35, 95% CI 0.06 to 2.18 | Very low |
| Ninan 2022 | Physical growth | Body weight (kg) (unadjusted) | Single course of ACS vs. no ACS | 2 studies, 2877 children | Preterm | 18 months to 22 months corrected age | MD 0.29, 95% CI - 1.1 to 0.51 | Very low |
| Ninan 2022 | Physical growth | Head circumference (cm) (unadjusted) | Single course of ACS vs. no ACS | 2 studies, 2817 children | Preterm | 18 months t0 22 months corrected age | MD 0.33, 95% CI -1.6 to 0.93 | Very low |
| Park 2016 | Neurodevelopmental | Neurologic impairment | Single course of ACS vs. placebo/no treatment | 1 study, 601 children | <24 weeks | 18 to 22 months | aOR 1.11, 95% CI 0.75 to 1.66 | Very low |
| Park 2016 | Neurodevelopmental | Neurologic impairment (unadjusted) | Single course of ACS vs. placebo/no treatment | 1 study, 68 children | 22 weeks | 18 to 22 months | OR 1.14, 95% CI 0.43 to 3.05 |  |
| Park 2016 | Neurodevelopmental | Neurologic impairment (unadjusted) | Single course of ACS vs. placebo/no treatment | 1 study, 533 children | 23 weeks | 18 to 22 months | OR 0.95, 95% CI 0.66 to 1.38 |  |
| Park 2016 | Neurodevelopmental | Neurologic impairment (unadjusted) | Single course of ACS vs. placebo/no treatment | 1 study, 601 children | <24 weeks | 18 to 22 months | OR 0.96, 95% CI 0.69 to 1.36 |  |
| Park 2016 | Survival/mortality | Mortality at 18-22 months | Single course of ACS vs. placebo/no treatment | 1 study | <24 weeks | 18 to 22 months | <24 weeks:  aOR 0.51, 95% CI 0.41 to 0.64  22 weeks:  aOR 0.60, 95% CI 0.43 to 1.07  23 weeks:  aOR 0.50, 95% CI 0.40 to 0.63 |  |
| Park 2016 | Survival/mortality | Mortality at 18-22 months (unadjusted) | Single course of ACS vs. placebo/no treatment | 1 study, 2251 children | <24 weeks | 18 to 22 months | OR 0.49, 95% CI 0.41 to 0.59 |  |
| Park 2016 | Survival/mortality | Mortality at 18-22 months (unadjusted) | Single course of ACS vs. placebo/no treatment | 1 study, 383 children | 22 weeks | 18 to 22 months | OR 0.59, 95% CI 0.35 to 0.98 |  |
| Park 2016 | Survival/mortality | Mortality at 18-22 months (unadjusted) | Single course of ACS vs. placebo/no treatment | 1 study, 1868 children | 22 weeks | 18 to 22 months | OR 0.52, 95% CI 0.43 to 0.64 |  |
| Park 2016 | Neurodevelopmental | Developmental delay –2 standard deviations or less (unadjusted) | Single course of ACS vs. placebo/no treatment | 1 study, 18 children | 23 weeks | 2 to 3 years | OR 1.29, 95% CI 0.07 to 24.38 |  |
| Park 2016 | Neurodevelopmental | Moderate to severe functional disability (unadjusted) | Single course of ACS vs. placebo/no treatment | 1 study, 23 children | 23 weeks | 2 to 3 years | OR 0.37, 95% CI 0.06 to 20.9 |  |
| Park 2016 | Neurodevelopmental | Mental developmental index <70 | Single course of ACS vs. placebo/no treatment | 1 study | <24 weeks | 18 to 22 months | <24 weeks: aOR 1.31, 95% CI 0.8 3 to 2.07 22 weeks:  aOR 2.16, 95% CI 0.36 to 13.10 23 weeks:  aOR 1.27, 95% CI 0.79 to 2.03 |  |
| Park 2016 | Neurodevelopmental | Mental developmental index <70 (unadjusted) | Single course of ACS vs. placebo/no treatment | 2 studies, 568 children | <24 weeks | 18 to 22 months | OR 1.35, 95% CI 0.81 to 1.65 |  |
| Park 2016 | Neurodevelopmental | Mental developmental index <70 (unadjusted) | Single course of ACS vs. placebo/no treatment | 1 study, 53 children | 22 weeks | 18 to 22 months | OR 2.00, 95% CI 0.64 to 6.26 |  |
| Park 2016 | Neurodevelopmental | Mental developmental index <70 (unadjusted) | Single course of ACS vs. placebo/no treatment | 2 studies, 515 children | 23 weeks | 18 to 22 months | OR 1.11, 95% CI 0.76 to 1.63 |  |
| Park 2016 | Neurodevelopmental | Psychomotor development index <70 | Single course of ACS vs. placebo/no treatment | 1 study | <24 weeks | 18 to 22 months | <24 weeks: aOR 1.00, 95% CI 0.65 to 1.54 22 weeks:  aOR 1.47, 95% CI 0.48 to 4.50 23 weeks:  aOR 0.93, 95% CI 0.58 to 1.49 |  |
| Park 2016 | Neurodevelopmental | Psychomotor development index <70 (unadjusted) | Single course of ACS vs. placebo/no treatment | 2 studies, 550 children | <24 weeks | 18 to 22 months | OR 1.23, 95% CI 0.57 to 2.64 |  |
| Park 2016 | Neurodevelopmental | Psychomotor development index <70 (unadjusted) | Single course of ACS vs. placebo/no treatment | 1 study, 55 children | 22 weeks | 18 to 22 months | OR 1.47, 95% CI 0.49 to 4.42 |  |
| Park 2016 | Neurodevelopmental | Psychomotor development index <70 (unadjusted) | Single course of ACS vs. placebo/no treatment | 2 studies, 495 children | 23 weeks | 18 to 22 months | OR 1.21, 95% CI 0.53 to 2.77 |  |
| Park 2016 | Neurodevelopmental | Bayley III cognitive score <70 | Single course of ACS vs. placebo/no treatment | 1 study | <24 weeks | 18 to 22 months | <24 weeks: aOR 0.37, 95% CI 0.12 to 1.11 22 weeks:  aOR 1.28, 95% CI 0.06 to 27.50 23 weeks:  aOR 0.31, 95% CI 0.10 to 1.00 |  |
| Park 2016 | Neurodevelopmental | Bayley III cognitive score <70 (unadjusted) | Single course of ACS vs. placebo/no treatment | 1 study, 114 children | <24 weeks | 18 to 22 months | OR 0.44, 95% CI 0.16 to 1.19 |  |
| Park 2016 | Neurodevelopmental | Bayley III cognitive score <70 (unadjusted) | Single course of ACS vs. placebo/no treatment | 1 study, 14 children | 22 weeks | 18 to 22 months | OR 1.10, 95% CI 0.07 to 28.12 |  |
| Park 2016 | Neurodevelopmental | Bayley III cognitive score <70 (unadjusted) | Single course of ACS vs. placebo/no treatment | 1 study, 100 children | 23 weeks | 18 to 22 months | OR 0.35, 95% CI 0.12 to 1.02 |  |
| Park 2016 | Neurodevelopmental | Moderate to severe cerebral palsy | Single course of ACS vs. placebo/no treatment | 1 study | <24 weeks | 18 to 22 months | <24 weeks: aOR 0.54, 95% CI 0.33 to 0.88 22 weeks:  aOR 0.22, 95% CI 0.23 to 3.34 23 weeks:  aOR 0.29, 95% CI 0.29 to 0.85 | - |
| Park 2016 | Neurodevelopmental | Moderate to severe cerebral palsy (unadjusted) | Single course of ACS vs. placebo/no treatment | 1 study, 619 children | <24 weeks | 18 to 22 months | OR 0.59, 95% CI 0.37 to 0.94 |  |
| Park 2016 | Neurodevelopmental | Moderate to severe cerebral palsy (unadjusted) | Single course of ACS vs. placebo/no treatment | 1 study, 72 children | 22 weeks | 18 to 22 months | OR 0.88, 95% CI 0.23 to 3.34 |  |
| Park 2016 | Neurodevelopmental | Moderate to severe cerebral palsy (unadjusted) | Single course of ACS vs. placebo/no treatment | 1 study, 547 children | 23 weeks | 18 to 22 months | OR 0.56, 95% CI 0.34 to 0.93 |  |
| Park 2016 | Neurodevelopmental | Blindness | Single course of ACS vs. placebo/no treatment | 1 study | 23 weeks | 18 to 22 months | aOR 0.31, 95% CI 0.10 to 0.93 |  |
| Park 2016 | Neurodevelopmental | Blindness (unadjusted) | Single course of ACS vs. placebo/no treatment | 1 study, 621 children | <24 weeks | 18 to 22 months | OR 0.36, 95% CI 0.13 to 0.95 |  |
| Park 2016 | Neurodevelopmental | Blindness (unadjusted) | Single course of ACS vs. placebo/no treatment | 1 study, 72 children | 22 weeks | 18 to 22 months | OR 0.30, 95% CI 0.01 to 6.45 |  |
| Park 2016 | Neurodevelopmental | Blindness (unadjusted) | Single course of ACS vs. placebo/no treatment | 1 study, 549 children | 23 weeks | 18 to 22 months | OR 0.38, 95% CI 0.14 to 1.07 |  |
| Park 2016 | Neurodevelopmental | Deafness | Single course of ACS vs. placebo/no treatment | 1 study | 23 weeks | 18 to 22 months | aOR 0.39, 95% CI 0.16 to 0.93 |  |
| Park 2016 | Neurodevelopmental | Deafness (unadjusted) | Single course of ACS vs. placebo/no treatment | 1 study, 615 children | <24 weeks | 18 to 22 months | OR 0.39, 95% CI 0.18 to 0.88 |  |
| Park 2016 | Neurodevelopmental | Deafness (unadjusted) | Single course of ACS vs. placebo/no treatment | 1 study, 70 children | 23 weeks | 18 to 22 months | OR 0.28, 95% CI 0.01 to 6.15 |  |
| Park 2016 | Neurodevelopmental | Deafness (unadjusted) | Single course of ACS vs. placebo/no treatment | 1 study, 545 children | 22 weeks | 18 to 22 months | OR 0.39, 95% CI 0.17 to 0.91 |  |
| Park 2016 | Respiratory | Chronic lung disease at 2 years (unadjusted) | Single course of ACS vs. placebo/no treatment | 1 study, 43 children | 23 weeks | 2 years | OR 1.05, 95% CI 0.22 to 4.98 |  |
| Sotiriadis 2015 | Neurodevelopmental | Cerebral palsy | Single course of ACS vs. placebo/no treatment (beta/dexa) | 7 studies, 6498 children | Mean GA 22 to 34 weeks | 18 months to 12 years | RR 0.678, 95% CI 0.564 to 0.815 |  |
| Sotiriadis 2015 | Neurodevelopmental | Cerebral palsy | Single course of ACS vs. placebo/no treatment (beta/dexa) | 4 studies, 5891 children | mean GA <28 weeks | 18 months to 5 years | RR 0.668, 95% CI 0.498 to 0.895 |  |
| Sotiriadis 2015 | Neurodevelopmental | Cerebral palsy | Single course of ACS vs. placebo/no treatment (beta/dexa) | 3 studies, 707 children | mean GA 28 to 34 weeks | 18 months to 12 years | RR 0.563, 95% CI 0.308 to 1.029 |  |
| Sotiriadis 2015 | Neurodevelopmental | Cerebral palsy | Single course of ACS vs. placebo/no treatment (beta) | 4 studies, 977 children | Mean GA 25 to 34 weeks | 18 months to 12 years | RR 0.828, 95% CI 0.491 to 1.396 |  |
| Sotiriadis 2015 | Neurodevelopmental | Cerebral palsy | Single course of ACS vs. placebo/no treatment (dexa) | 3 studies, 1049 children | Mean GA 26 to 34 weeks | 18 to 36 months | RR 0.726, 95% CI 0.435 to 1.211 |  |
| Sotiriadis 2015 | Neurodevelopmental | Cerebral palsy | Single course of ACS vs. placebo/no treatment (beta/dexa, RCTs only) | 3 studies, 707 children | Mean GA 28 to 34 weeks | 18 months to 12 years | RR 0.563, 95% CI 0.308 to 1.029 |  |
| Sotiriadis 2015 | Neurodevelopmental | Severe Disability | Single course of ACS vs. placebo/no treatment (beta/dexa) | 5 studies, 6051 children | Mean GA 22 to 34 weeks | 18 months to 12 years | RR 0.787, 95% CI 0.729 to 0.850 |  |
| Sotiriadis 2015 | Neurodevelopmental | Severe Disability | Single course of ACS vs. placebo/no treatment (beta/dexa) | 3 studies, 5818 children | mean GA <28 weeks | 18 to 36 moths | RR 0.785, 95% CI 0.726 to 0.848 |  |
| Sotiriadis 2015 | Neurodevelopmental | Severe disability | Single course of ACS vs. placebo/no treatment (beta/dexa) | 2 studies, 386 children | mean GA 28 to 34 weeks | 2 to 12 years | RR 0.875, 95% CI 0.545 to 1.403 |  |
| Sotiriadis 2015 | Neurodevelopmental | Severe Disability | Single course of ACS vs. placebo/no treatment (beta) | 3 studies, 1079 children | Mean GA 26 to 34 weeks | 18 to 24 months | RR 0.776, 95% CI 0.642 to 0.937 |  |
| Sotiriadis 2015 | Neurodevelopmental | Severe Disability | Single course of ACS vs. placebo/no treatment (dexa) | 2 studies, 643 children | Mean GA 26 to 34 weeks | 18 months to 12 years | RR 0.902, 95% CI 0.732 to 1.111 |  |
| Sotiriadis 2015 | Neurodevelopmental | Severe disability | Single course of ACS vs. placebo/no treatment (dexa, RCTs only) | 1 study, 82 children | Mean GA 24 to 34 weeks | 11 to 12 years | RR 0.563, 95% CI 0.308 to 1.029 |  |
| Sotiriadis 2015 | Neurodevelopmental | Mental development index <70 | Single course of ACS vs. placebo/no treatment (beta/dexa) | 4 studies, 4939 children | Mean GA 22 to 34 weeks | 18 to 36 months | RR 0.840, 95% CI 0.692 to 1.020 |  |
| Sotiriadis 2015 | Neurodevelopmental | Mental development index <70 | Single course of ACS vs. placebo/no treatment (beta/dexa) | 3 studies, 4509 children | Mean GA <28 weeks | 18 to 36 months | RR 0.806, 95% CI 0.596 to 1.090 |  |
| Sotiriadis 2015 | Neurodevelopmental | Mental development index <70 | Single course of ACS vs. placebo/no treatment (dexa) | 2 studies, 991 children | Mean GA 26 to 34 weeks | 18 to 36 months | RR 0.853, 95% CI (0.673–1.079) |  |
| Sotiriadis 2015 | Neurodevelopmental | Mental developmental index <70 | Single course of ACS vs. placebo/no treatment (beta) | 2 studies, 775 children | <28 weeks | 18 to 22 months | RR 0.762, 95% CI 0.603 to 0.963 |  |
| Sotiriadis 2015 | Neurodevelopmental | Psychomotor development <70 | Single course of ACS vs. placebo/no treatment (beta/dexa) | 2 studies, 4018 children | Mean GA 22 to 34 weeks | 18 to 36 months | RR 0.829, 95% CI 0.737 to 0.933 |  |
| Sotiriadis 2015 | Neurodevelopmental | Psychomotor development <70 | Single course of ACS vs. placebo/no treatment (dexa) | 2 studies, 985 children | Mean GA 26 to 34 weeks | 18 to 36 months | RR 0.984, 95% CI 0.553 to 1.753 |  |
| Sotiriadis 2015 | Neurodevelopmental | Intact survival | Single course of ACS vs. placebo/no treatment (beta/dexa) | 6 studies, 2644 children | Mean GA 25 to 34 weeks | 18 months to 3 years | RR 1.186, 95% CI 1.056 to 1.332 |  |
| Sotiriadis 2015 | Neurodevelopmental | Intact survival | Single course of ACS vs. placebo/no treatment (beta/dexa) | 3 studies, 2158 children | <28 weeks | 18 to 36 months | RR 1.377, 95% CI 0.934 to 2.029 |  |
| Sotiriadis 2015 | Neurodevelopmental | Intact survival | Single course of ACS vs. placebo/no treatment (beta/dexa) | 3 studies, 486 children | Mean GA 28 to 34 weeks | 24 months | RR 1.148, 95% CI 1.036 to 1.273 |  |
| Sotiriadis 2015 | Neurodevelopmental | Intact survival | Single course of ACS vs. placebo/no treatment (beta) | 4 studies, 1213 children | Mean GA between 25 and 34 weeks | 24 to 36 months | RR 1.329, 95% CI 1.047 to 1.686 |  |
| Sotiriadis 2015 | Neurodevelopmental | Intact survival | Single course of ACS vs. placebo/no treatment (dexa) | 2 studies, 643 children | Mean GA between 26 and 34 weeks | 24 to 36 months | RR 1.329, 95% CI 1.047 to 1.686 |  |
| Sotiriadis 2015 | Neurodevelopmental | Minor disability | Single course of ACS vs. placebo/no treatment (beta/dexa) | 3 studies, 1327 children | Mean GA 26 to 34 weeks | 24 to 36 months | RR 0.826, 95% CI 0.638 to 1.068 |  |
| Sotiriadis 2015 | Neurodevelopmental | Minor disability | Single course of ACS vs. placebo/no treatment (beta/dexa) | 2 studies, 386 children | Mean GA 28 to 34 weeks | 24 months | RR 0.754, 95% CI 0.547 to 1.038 |  |
| Crowley 1995 | Neurodevelopmental | Neurologic abnormality | Any ACS vs. placebo | 3 trials, 778 children |  | 2 to 12 years | OR 0.62 |  |
| **Any ACS exposure (single/multiple/repeat/unspecified courses) vs. placebo/no ACS** | | | | | | | | |
| Wang 2022 | Neurodevelopmental | Hearing impairment | ACS exposure vs. no ACS exposure | 7 studies, 8130 children | <34 weeks | up to 3 years | OR 0.64, 95% CI 0.48 to 0.87 |  |
| Ninan 2022 | Neurodevelopmental | Neurodevelopmental impairment | Unspecified no. of ACS course vs. no ACS | 5 studies | Preterm | 1 to 3 years | aOR 0.78, 95% CI 0.57 to 1.06 | Low |
| Ninan 2022 | Neurodevelopmental | Neurodevelopmental impairment (unadjusted) | Unspecified no. of ACS course vs. no ACS | 5 studies, 2722 children | Preterm | 12 months to 42 months | OR 0.96, 95% CI 0.61 to 1.50 | Very low |
| Ninan 2022 | Neurodevelopmental | Cerebral palsy (unadjusted) | Unspecified no. of ACS course vs. no ACS | 3 studies | Preterm | 18 months to 36 months | RR 0.86, 95% CI 0.76 to 0.98 | Low |
| Ninan 2022 | Neurodevelopmental | Auditory impairment | Unspecified no. of ACS course vs. no ACS | 2 studies, 10567 children | Preterm | 18 months to 36 months | ORR 0.77, 95% CI 0.36 to 0.1.77 | Very low |
| Ninan 2022 | Neurodevelopmental | Visual impairment (unadjusted) | Unspecified no. of ACS course vs. no ACS | 2 studies, 7990 children | Preterm | 18 months to 36 months | RR 1.15, 95 CI 0.96 to 1.35 | Very low |
| Amiya 2016 | Neurodevelopmental | Cerebral palsy | Single/repeat courses of ACS vs. placebo/no ACS (Dexa) in women with intrapartum bacterial infections | 1 study, 72 children | <30 weeks | 1 to 3 years | OR 0.35, 95% CI 0.07 to 1.67 | Very low |
| Amiya 2016 | Neurodevelopmental | General Development Quotient at 1 years' follow up | Single/repeat courses of ACS vs. placebo/no ACS (Dexa) in women with chorioamnionitis | 1 study, 72 children | <30 weeks | 1 year | MD 6.00, 95% CI - 8.94 to 20.94 | Very low |
| Amiya 2016 | Neurodevelopmental | General Development Quotient at 3 years' follow up | Single/repeat courses of ACS vs. placebo/no ACS (Dexa) in women with chorioamnionitis | 1 study, 72 children | <30 weeks | 3 years | MD 13.00, 95% CI - 3.75 to 29.75 | Very low |
| Amiya 2016 | Physical growth | Growth <10th percentile in early childhood | Single/repeat courses of ACS vs. placebo/no ACS (Beta) in women with growth-restricted fetus | 1 study, 91 children | 26 to 31 weeks | early childhood | OR 5.20, 95% CI 1.38 to 19.62 | Low |
| **Repeat/multiple courses of ACS vs. single course of ACS** | | | | | | | | |
| Walters 2022 | Neurodevelopmental | Neurodevelopmental impairment at early childhood follow up | Repeat doses of ACS vs. single course of ACS | 4 studies, 3616 children | Preterm or at term | 2 to 3 years | RR 0.97, 95% CI 0.85 to 1.10 | High |
| Walters 2022 | Neurodevelopmental | Cerebral palsy at early childhood follow-up | Repeat doses of ACS vs. single course of ACS | 5 studies, 3923 children | Preterm or at term | 2 to <5 years | RR 1.03, 95% CI 0.71, 1.49 | Moderate |
| Walters 2022 | Neurodevelopmental | Developmental delay or intellectual impairment at early childhood follow-up | Repeat doses of ACS vs. single course of ACS | 4 studies, 3581 children | Preterm or at term | 2 to <5 years | RR 0.95, 95% CI 0.84, 1.09 | High |
| Walters 2022 | Neurodevelopmental | Mental Developmental Index at early childhood follow-up | Repeat doses of ACS vs. single course of ACS | 3 studies, 1627 children | Preterm or at term | 2 to <5 years | MD 0.89, 95% CI -0.61 to 2.39 | High |
| Walters 2022 | Psychological | Child behaviour at early childhood follow-up, Child Behaviour Checklist total score in the clinical range | Repeat doses of ACS vs. single course of ACS | 1 study, 1045 children | Preterm or at term | 2 years | RR 1.09, 95% CI 0.79 to 1.51 | Moderate |
| Walters 2022 | Psychological | Child behaviour: Behaviour rating scale in the clinical range (BSID-II) | Repeat doses of ACS vs. single course of ACS | 1 study, 1776 children | Preterm or at term | 2 to <5 years | RR 1.16, 95% CI 1.00 to 1.34 | Moderate |
| Walters 2022 | Psychological | Child behaviour at early childhood follow-up as assessed by Early Child Behaviour Questionnaire Extraversion summary scale | Repeat doses of ACS vs. single course of ACS | 1 study, 142 children | Preterm | 2 years | MD 0.00, 95% CI -0.18 to 0.18 | Moderate |
| Walters 2022 | Psychological | Child behaviour at early childhood follow-up assessed by Early Child Behaviour Questionnaire Negative affectivity summary scale | Repeat doses of ACS vs. single course of ACS | 1 study, 142 children | Preterm | 2 years | MD -0.10, 95% CI -0.27 to 0.07 | Moderate |
| Walters 2022 | Psychological | Child behaviour at early childhood follow-up assessed by Early Child Behaviour Questionnaire Effortful control summary scale | Repeat doses of ACS vs. single course of ACS | 1 study, 142 children | Preterm | 2 years | MD 0.00, 95% CI -0.17 to 0.17 | Moderate |
| Walters 2022 | Neurodevelopmental | Psychomotor Developmental Index at early childhood follow-up | Repeat doses of ACS vs. single course of ACS | 2 studies, 1423 children | Preterm or at term | 2 to <5 years | MD 1.26, 95% CI -0.45 to 2.96 | Moderate |
| Walters 2022 | Neurodevelopmental | Deafness/hearing impairment at early childhood follow-up | Repeat doses of ACS vs. single course of ACS | 4 studies, 3528 children | Preterm or at term | 2 to <5 years | RR 0.97, 95% CI 0.56 to 1.71 | Moderate |
| Walters 2022 | Neurodevelopmental | Blindness/visual impairment at early childhood follow-up | Repeat doses of ACS vs. single course of ACS | 3 studies, 3274 children | Preterm or at term | 2 to <5 years | RR 1.17, 95% CI 0.65 to 2.10 | Moderate |
| Walters 2022 | Respiratory | Asthma or recurrent wheeze at early childhood follow-up | Repeat doses of ACS vs. single course of ACS | 3 studies, 1720 children | Preterm or at term | 2 to <5 years | RR 0.89, 95% CI 0.74 to 1.06 | Moderate |
| Walters 2022 | Respiratory | Any respiratory disease at early childhood follow-up | Repeat doses of ACS vs. single course of ACS | 3 studies, 3423 children | Preterm or at term | 2 to <5 years | RR 1.04, 95% CI 0.92, 1.18 | High |
| Walters 2022 | Physical growth | Mean weight at early childhood follow-up (kg) | Repeat doses of ACS vs. single course of ACS | 4 studies, 3784 children | Preterm or at term | 2 to <5 years | MD - 0.16, 95% CI -0.25 to -0.07 | High |
| Walters 2022 | Physical growth | Mean weight z-score at early childhood | Repeat doses of ACS vs. single course of ACS | 1 study, 1047 children | Preterm or at term | 2 years | MD -0.03, 95% CI -0.19 to 0.13 | High |
| Walters 2022 | Physical growth | Mean weight adjusted for age at early childhood follow‐up (standardised mean difference) | Repeat doses of ACS vs. single course of ACS | 3 studies, 1776 children | Preterm or at term | 2 to <5 years | SMD ‐0.06, 95% CI ‐0.15 to 0.04 | High |
| Walters 2022 | Physical growth | Weight small for age at early childhood follow-up | Repeat doses of ACS vs. single course of ACS | 2 studies, 1533 children | Preterm or at term | 2 to <5 years | RR 0.92, 95% CI 0.72 to 1.19 | Moderate |
| Walters 2022 | Physical growth | Mean head circumference at early childhood follow-up (cm) | Repeat doses of ACS vs. single course of ACS | 4 studies, 3784 children | Preterm or at term | 2 to <5 years | MD -0.06, 95% CI 0.18 to 0.06 | High |
| Walters 2022 | Physical growth | Mean head circumference Z score at early childhood follow‐up | Repeat doses of ACS vs. single course of ACS | 2 studies, 1290 children | Preterm or at term | 2 to <5 years | MD 0.04, 95% CI ‐0.09 to 0.18 | High |
| Walters 2022 | Physical growth | Head circumference small for age at early childhood follow-up | Repeat doses of ACS vs. single course of ACS | 2 studies, 1527 children | Preterm or at term | 2 to <5 years | RR 1.02, 95% CI 0.87 to 1.21 | Moderate |
| Walters 2022 | Physical growth | Mean height at early childhood follow-up (cm) | Repeat doses of ACS vs. single course of ACS | 4 studies, 3784 children | Preterm or at term | 2 to <5 years | MD -0.07, 95% CI -0.31 to 0.17 | High |
| Walters 2022 | Physical growth | Height Z score at early childhood follow‐up | Repeat doses of ACS vs. single course of ACS | 2 studies, 1290 children | Preterm or at term | 2 to <5 years | MD - 0.40, 95% CI -0.17 to 0.09 | High |
| Walters 2022 | Physical growth | Mean height adjusted for age at early childhood follow‐up (standardised mean difference) | Repeat doses of ACS vs. single course of ACS | 3 studies, 1776 children | Preterm or at term | 2 to <5 years | SMD -0.06, 95% CI -0.15 to 0.04 | High |
| Walters 2022 | Physical growth | Height small for age at early childhood follow-up | Repeat doses of ACS vs. single course of ACS | 2 studies, 1526 children | Preterm or at term | 2 to <5 years | RR 1.03, 95% CI 0.77 to 1.36 | Low |
| Walters 2022 | Cardiovascular | Mean systolic blood pressure at early childhood follow-up (mmHg) | Repeat doses of ACS vs. single course of ACS | 1 study, 486 children | Preterm or at term | 2 to <5 years | MD -2.90, 95% CI -5.4 to -0.40 | High |
| Walters 2022 | Cardiovascular | Mean systolic blood pressure Z score at early childhood follow-up (mmHg) | Repeat doses of ACS vs. single course of ACS | 1 study, 672 children | Preterm or at term | 2 to <5 years | MD -0.10, 95% CI -0.28 to 0.08 | Moderate |
| Walters 2022 | Cardiovascular | Mean diastolic blood pressure at early childhood follow-up (mmHg) | Repeat doses of ACS vs. single course of ACS | 1 study, 486 children | Preterm or at term | 2 to <5 years | MD -1.00, 95% CI -2.86 to 0.86 | High |
| Walters 2022 | Cardiovascular | Mean diastolic blood pressure Z score at early childhood follow-up (mmHg) | Repeat doses of ACS vs. single course of ACS | 1 study, 628 children | Preterm or at term | 2 to <5 years | MD 0.10, 95% CI -0.05 to 0.25 | Moderate |
| Walters 2022 | Cardiovascular | Hypertension at early childhood follow-up | Repeat doses of ACS vs. single course of ACS | 1 study, 628 children | Preterm or at term | 2 years | RR 0.97, 95% CI 0.77 to 1.23 | Moderate |
| Walters 2022 | Neurodevelopmental | Survival free of neurodevelopmental impairment at early childhood follow up | Repeat doses of ACS vs. single course of ACS | 4 studies, 3845 children | Preterm or at term | 2 to 3 years | RR 1.01, 95% CI 0.98 to 1.04 | High |
| Walters 2022 | Neurodevelopmental | Survival free of major neurodevelopmental impairment at early childhood follow up | Repeat doses of ACS vs. single course of ACS | 3 studies, 1816 children | Preterm or at term | 2 to 3 years | RR 1.02, 95% CI 0.98 to 1.05 | Moderate |
| Walters 2022 | Neurodevelopmental | Survival free of neurocognitive impairment at mid- to later childhood follow up | Repeat doses of ACS vs. single course of ACS | 1 study, 963 children | Preterm and term | 5 to 8 years | RR 1.0, 95% CI 0.95 to 1.08 | Moderate |
| Walters 2022 | Neurodevelopmental | Survival free of major neurocognitive impairment at mid- to later childhood follow up | Repeat doses of ACS vs. single course of ACS | 2 studies, 2682 children | Preterm and term | 5 to 8 years | RR 1.00, 95% CI 0.97 to 1.04 | Moderate |
| Walters 2022 | Neurodevelopmental | Neurocognitive impairment at mid- to later childhood follow up | Repeat doses of ACS vs. single course of ACS | 1 study, 897 children | Preterm and term | 5 to 8 years | RR 0.96, 95% CI 0.72 to 1.29 | Low |
| Walters 2022 | Neurodevelopmental | Cognitive impairment at mid- to later childhood follow-up | Repeat doses of ACS vs. single course of ACS | 2 studies, 2504 children | Preterm and term | 5 to 8 years | RR 1.00, 95% CI 0.81 to 1.22 | Moderate |
| Walters 2022 | Neurodevelopmental | Cerebral palsy at mid- to later childhood follow-up | Repeat doses of ACS vs. single course of ACS | 2 studies, 2622 children | Preterm and term | 5 to 8 years | RR 0.74, 95% CI 0.43 to 1.26 | Low |
| Walters 2022 | Neurodevelopmental | Deafness/hearing impairment at mid- to later childhood follow-up (2 studies) | Repeat doses of ACS vs. single course of ACS | 2 studies, 2532 children | Preterm and term | 5 to 8 years | RR 1.62, 95% CI 0.77 to 3.41 | Low |
| Walters 2022 | Neurodevelopmental | Blindness/visual impairment at mid- to later childhood follow-up (2 studies) | Repeat doses of ACS vs. single course of ACS | 2 studies, 2532 children | Preterm and term | 5 to 8 years | RR 1.12, 95% CI 0.79 to 1.59 | Low |
| Walters 2022 | Psychological | Abnormal child behaviour at mid- to later childhood follow-up | Repeat doses of ACS vs. single course of ACS | 1 study, 1615 children | Preterm and term | 5 years | RR 0.96 95% CI 0.75 to 1.22 | Moderate |
| Walters 2022 | Psychological | Child behaviour at mid- to later childhood follow-up (standardised mean difference) | Repeat doses of ACS vs. single course of ACS | 2 studies, 2480 children | Preterm and term | 5 to 8 years | MD -0.00, 95% CI -0.08 to 0.08 | Moderate |
| Walters 2022 | Respiratory | Asthma or recurrent wheeze at mid- to later childhood follow-up | Repeat doses of ACS vs. single course of ACS | 1 study, 979 children | Preterm and term | 6 to 8 years | RR 1.0, 95% CI 0.85 to 1.19 | High |
| Walters 2022 | Respiratory | Any respiratory disease at mid- to later childhood follow-up | Repeat doses of ACS vs. single course of ACS | 1 study, 979 children | Preterm and term | 6 to 8 years | RR 0.79, 95% CI 0.36 to 1.72 | Very low |
| Walters 2022 | Physical growth | Mean weight at mid- to later childhood follow-up (kg) | Repeat doses of ACS vs. single course of ACS | 1 study, 1635 children | Preterm and term | 5 years | MD -0.20, 95% CI -0.62 to 0.22 | Moderate |
| Walters 2022 | Physical growth | Mean weight Z score at mid- to later childhood follow-up | Repeat doses of ACS vs. single course of ACS | 1 study, 940 children | Preterm and term | 6 to 8 years | MD -0.06, 95% CI -0.23 to 0.11 | High |
| Walters 2022 | Physical growth | Mean head circumference at mid- to later childhood follow-up (cm) | Repeat doses of ACS vs. single course of ACS | 1 study, 1635 children | Preterm and term | 5 years | MD -0.10, 95% CI -0.36 to 0.16 | Moderate |
| Walters 2022 | Physical growth | Head circumference Z score at mid- to later childhood follow-up | Repeat doses of ACS vs. single course of ACS | 1 study, 885 children | Preterm and term | 6 to 8 years | MD -0.04, 95% CI -0.22 to 0.14 | Moderate |
| Walters 2022 | Physical growth | Mean height at mid- to later childhood follow-up (cm) | Repeat doses of ACS vs. single course of ACS | 1 study, 1635 children | Preterm and term | 5 years | MD -0.40, 95% CI -1.11 to 0.31 | Moderate |
| Walters 2022 | Physical growth | Mean height Z score at mid- to later childhood follow-up | Repeat doses of ACS vs. single course of ACS | 1 study, 912 children | Preterm and term | 6 to 8 years | MD 0.02, 95% CI -0.13 to 0.17 | High |
| Walters 2022 | Physical growth | BMI Z scores at mid- to later childhood follow-up | Repeat doses of ACS vs. single course of ACS | 1 study, 910 children | Preterm and term | 6 to 8 years | MD -0.13, 95% CI -0.30 to 0.04 | High |
| Walters 2022 | Physical growth | Body composition: total body fat-free mass at mid- to later childhood follow-up (kg) | Repeat doses of ACS vs. single course of ACS | 1 study, 185 children | Preterm and term | 6 to 8 years | MD -0.20, 95% CI -1.43 to 1.03 |  |
| Walters 2022 | Physical growth | Body composition: total body fat mass at mid- to later childhood follow-up (kg) | Repeat doses of ACS vs. single course of ACS | 1 study, 185 children | Preterm and term | 6 to 8 years | MD 0.10, 95% CI -0.71 to 0.91 |  |
| Walters 2022 | Cardiovascular | Mean systolic blood pressure at mid- to later childhood follow-up (mmHg) | Repeat doses of ACS vs. single course of ACS | 1 study, 1635 children | Preterm and term | 5 years | MD 0.30, 95% CI -1.06 to 1.66 | Moderate |
| Walters 2022 | Cardiovascular | Mean diastolic blood pressure at mid- to later childhood follow-up (mmHg) | Repeat doses of ACS vs. single course of ACS | 1 study, 1635 children | Preterm and term | 5 years | MD 0.70, 95% CI -0.46 to 1.86 | Moderate |
|  | Cardiovascular | Mean systolic blood pressure Z score at mid- to later childhood follow-up | Repeat doses of ACS vs. single course of ACS | 1 study, 848 children | Preterm or at term | 6 to 8 years | MD 0.07, 95% CI -0.06 to 0.20 | Moderate |
| Walters 2022 | Cardiovascular | Mean diastolic blood pressure Z score at mid- to later childhood follow-up | Repeat doses of ACS vs. single course of ACS | 1 study, 848 children | Preterm or at term | 6 to 8 years | MD -0.09, 95% CI -0.25 to 0.07 | Moderate |
| Walters 2022 | Respiratory | Measures of lung function at mid- to later childhood follow-up: mean FEV1 Z score | Repeat doses of ACS vs. single course of ACS | 1 study, 185 children | Preterm or at term | 6 to 8 years | MD -0.06, 95% CI -0.34 to 0.22 | Very low |
| Walters 2022 | Respiratory | Measures of lung function at mid- to later childhood follow-up: mean FVC Z score | Repeat doses of ACS vs. single course of ACS | 1 study, 185 children | Preterm or at term | 6 to 8 years | MD -0.18, 95% CI -0.49 to 0.13 | Very low |
| Walters 2022 | Respiratory | Measures of lung function at mid- to later childhood follow-up: mean FEV1/FVC Z score | Repeat doses of ACS vs. single course of ACS | 1 study, 185 children | Preterm or at term | 6 to 8 years | MD -0.19, 95% CI -0.44 to 0.06 | Very low |
| Crowther 2019 | Neurodevelopmental | Death or neurosensory disability | Repeat doses of ACS vs. single course of ACS | 6 studies, 4557 children | Preterm or at term | 2 to 3 years | RR 1.03, 95% CI 0.94 to 1.13 |  |
| Crowther 2019 | Neurodevelopmental | Cerebral palsy | Repeat doses of ACS vs. single course of ACS | 5 studies, 3911 children |  |  | RR 1.02, 95% CI 0.70 to 1.49 |  |
| Crowther 2019 | Neurodevelopmental | Developmental delay or intellectual impairment at early childhood follow-up | Repeat doses of ACS vs. single course of ACS | 4 studies, 3652 children |  |  | RR 1.02, 95% CI 0.92 to 1.14 |  |
| Crowther 2019 | Neurodevelopmental | Blindness | Repeat doses of ACS vs. single course of ACS | 4 studies, 3045 children |  |  | RR 1.14, 95% CI 0.62 to 2.12 |  |
| Crowther 2019 | Neurodevelopmental | Deafness | Repeat doses of ACS vs. single course of ACS | 4 studies, 3041 children |  |  | RR 1.02, 95% CI 0.57 to 1.85 |  |
| Crowther 2019 | Neurodevelopmental | Gross motor dysfunction | Repeat doses of ACS vs. single course of ACS | 4 studies, 3727 children |  |  | RR 0.98, 95% CI 0.74 to 1.29 |  |
| Crowther 2019 | Neurodevelopmental | Motor delay | Repeat doses of ACS vs. single course of ACS | 4 studies, 3559 children |  |  | RR 1.05, 95% CI 0.92 to 1.21 |  |
| Crowther 2019 | Neurodevelopmental | Any neurosensory disability | Repeat doses of ACS vs. single course of ACS | 5 studies, 4125 children |  |  | RR 1.03, 95% CI 0.93 to 1.14 |  |
| Crowther 2019 | Neurodevelopmental | Major neurosensory disability | Repeat doses of ACS vs. single course of ACS | 5 studies, 4002 children |  |  | RR 1.03, 95% CI 0.85 to 1.25 |  |
| Crowther 2019 | Physical growth | Weight at follow up (z-scores) | Repeat doses of ACS vs. single course of ACS | 4 studies, 3734 children |  |  | MD -0.11, 95% CI - 0.19 to -0.03 |  |
| Crowther 2019 | Physical growth | Head circumference at follow up (z-score) | Repeat doses of ACS vs. single course of ACS | 4 studies, 3657 children |  |  | MD -0.07, 95% CI -0.15 to 0.02 |  |
| Crowther 2019 | Physical growth | Height at follow up (Z-score) | Repeat doses of ACS vs. single course of ACS | 4 studies, 3410 children | - |  | MD -0.08, 95% CI -0.18 to 0.01 |  |
| Crowther 2019 | Psychological | Child behaviour | Repeat doses of ACS vs. single course of ACS | 2 studies, 1432 children |  |  | RR 1.11, 95% CI 0.92 to 1.35 |  |
| Crowther 2019 | Respiratory | Respiratory disease | Repeat doses of ACS vs. single course of ACS | 4 studies, 3890 children |  |  | RR 0.96, 95% CI 0.86 to 1.08 |  |
| Crowther 2019 | Cardiovascular | Follow up blood pressure (systolic) (mmHg) | Repeat doses of ACS vs. single course of ACS | 2 studies, 1101 children |  |  | MD -1.73, 95% CI -3.39 to -0.08 |  |
| Crowther 2019 | Cardiovascular | Follow up blood pressure (diastolic) (mmHg) | Repeat doses of ACS vs. single course of ACS | 2 studies, 1066 children |  |  | MD -0.48, 95% CI -1.81 to 0.84 |  |
| Crowther 2019 | Cardiovascular | Follow up blood pressure (mean arterial) (mmHg) | Repeat doses of ACS vs. single course of ACS | 2 studies, 1056 children |  |  | MD -0.80, 95% CI -2.13 to 0.53 |  |
| Peltoniemi 2011 | Neurodevelopmental | Death or severe neurological impairment | Repeat doses of ACS vs. single course of ACS | 4 studies, 3421 children | Preterm or at term | 2 years | RR 0.98, 95% CI 0.79 to 1.20 |  |
| Peltoniemi 2011 | Neurodevelopmental | Cerebral palsy | Repeat doses of ACS vs. single course of ACS | 4 studies, 3811 children | Preterm or at term | 2 years | RR 0.99, 95% CI 0.68 to 1.45 |  |
| Peltoniemi 2011 | Physical growth | Weight (kg) | Repeat doses of ACS vs. single course of ACS | 4 studies, 3797 children | Preterm or at term | 2 years | MD - 0.058, 95% CI -0.122 to 0.005 |  |
| Peltoniemi 2011 | Physical growth | Height (cm) | Repeat doses of ACS vs. single course of ACS | 4 studies, 3797 children | Preterm or at term | 2 years | MD - 0.021, 95% CI - 0.084 to 0.043 |  |
| Peltoniemi 2011 | Physical growth | Head circumference (cm) | Repeat doses of ACS vs. single course of ACS | 4 studies, 3797 children | Preterm or at term | 2 years | MD - 0.032, 95% CI - 0.096 to 0.032 |  |
| **ACS vs. ACS** | | | | | | | | |
| Williams 2022 | Neurodevelopmental | Neurodevelopmental disability at follow up | Dexa vs. Beta | 2 studies, 1151 children | Preterm and at term | 18 to 24 months | RR 1.02, 95% CI 0.85 to 1.22 | Moderate |
| Williams 2022 | Neurodevelopmental | Visual impairment | Dexa vs. Beta | 1 study, 1227 children | Mean GA (dexa: 34.7 weeks, beta: 34.5 weeks) | 2 years | RR 0.33, 95% CI 0.01 to 8.15 | Low |
| Williams 2022 | Neurodevelopmental | Hearing impairment | Dexa vs. Beta | 1 study, 1227 children | Mean GA (dexa: 34.7 weeks, beta: 34.5 weeks) | 2 years | RR 1.16, 95% CI 0.63 to 2.16 | Moderate |
| Williams 2022 | Neurodevelopmental | Intellectual impairment: cognitive or language developmental delay (mild, moderate or severe) | Dexa vs. Beta | 1 study, 1161 children | Mean GA (dexa: 34.7 weeks, beta: 34.5 weeks) | 2 years | RR 0.97, 95% CI 0.79 to 1.20 | Moderate |
| Williams 2022 | Neurodevelopmental | Developmental delay – motor (mild, moderate or severe) | Dexa vs. Beta | 1 study, 1166 children | Mean GA (dexa: 34.7 weeks, beta: 34.5 weeks) | 2 years | RR 0.89, 95% CI 0.66 to 1.20 | Moderate |
| Williams 2022 | Survival/mortality | Death in childhood (death after hospital discharge) | Dexa vs. Beta | 1 study, 1509 children | Mean GA (dexa: 34.7 weeks, beta: 34.5 weeks) | 2 years | RR 0.65, 95% CI 0.11 to 3.89 |  |
| Williams 2022 | Neurodevelopmental | Developmental delay – motor (moderate or severe) | Dexa vs. Beta | 1 study, 1166 children | Mean GA (dexa: 34.7 weeks, beta: 34.5 weeks) | 2 years | RR 0.84, 95% CI 0.45 to 1.56 |  |
| Williams 2022 | Neurodevelopmental | Intellectual impairment: cognitive or language developmental delay (moderate or severe) | Dexa vs. Beta | 1 study, 1161 children | Mean GA (dexa: 34.7 weeks, beta: 34.5 weeks) | 2 years | RR 1.23, 95% CI 0.80 to 1.90 |  |
| Williams 2022 | Neurodevelopmental | Cerebral palsy | Dexa vs. Beta | 1 study, 1223 children | Mean GA (dexa: 34.7 weeks, beta: 34.5 weeks) | 2 years | RR 2.50, 95% CI 0.97 to 6.39 | Low |
| Williams 2022 | Physical growth | Mean weight (kg) | Dexa vs. Beta | 1 study, 1208 children | Mean GA (dexa: 34.7 weeks, beta: 34.5 weeks) | 2 years | MD 0.00, 95% CI -0.23 to 0.23 |  |
| Williams 2022 | Physical growth | Mean height (cm) | Dexa vs. Beta | 1 study, 1187 children | Mean GA (dexa: 34.7 weeks, beta: 34.5 weeks) | 2 years | MD 0.20, 95% CI -0.39 to 0.79 |  |
| Williams 2022 | Physical growth | Mean head circumference (cm) | Dexa vs. Beta | 1 study, 1188 children | Mean GA (dexa: 34.7 weeks, beta: 34.5 weeks) | 2 years | MD 0.10, 955 CI -0.14 to 0.34 |  |
| Williams 2022 | Cardiovascular | Mean systolic blood pressure (mmHg) | Dexa vs. Beta | 1 study, 724 children | Mean GA (dexa: 34.7 weeks, beta: 34.5 weeks) | 2 years | MD -0.60, 95% CI -2.23 to 1.03 |  |
| Williams 2022 | Cardiovascular | Mean diastolic blood pressure (mmHg) | Dexa vs. Beta | 1 study, 703 children | Mean GA (dexa: 34.7 weeks, beta: 34.5 weeks) | 2 years | MD -0.10, 95% CI -1.67 to 1.47 |  |
| Williams 2022 | Psychological | Childhood behaviour checklist (mean total score) | Dexa vs. Beta | 1 study, 1253 children | Mean GA (dexa: 34.7 weeks, beta: 34.5 weeks) | 2 years | MD -0.40, 95% CI -2.68 to 1.88 |  |
| Williams 2022 | Neurodevelopmental | Educational achievement – cognitive score (mean, BSID-III) | Dexa vs. Beta | 1 study, 1199 children | Mean GA (dexa: 34.7 weeks, beta: 34.5 weeks) | 2 years | MD 0.50, 95% CI -1.28 to 2.28 |  |
| Williams 2022 | Neurodevelopmental | Educational achievement – language score (mean, BSID-III) | Dexa vs. Beta | 1 study, 1160 children | Mean GA (dexa: 34.7 weeks, beta: 34.5 weeks) | 2 years | MD 0.30, 95% CI -1.81 to 2.41 |  |
| Williams 2022 | Neurodevelopmental | Educational achievement – motor score (mean, BSID-III) | Dexa vs. Beta | 1 study, 1160 children | Mean GA (dexa: 34.7 weeks, beta: 34.5 weeks) | 2 years | MD 0.10, 95% CI -1.75 to 1.95 |  |
| Ciapponi 2021 | Neurodevelopmental | Neurodevelopmental disability | Dexa vs. Beta | 2 studies, 1151 children | Preterm and at term | 18 to 24 months | OR 1.03, 95% CrI 0.8 to 1.33 | Moderate |

RR= Relative risk; MD= Mean difference; SMD= Standardized mean difference; OR= odds ratio; aOR= adjusted odds ratio
