# Supplementary material for: Long-term outcomes of antenatal corticosteroids for preterm birth: An overview of systematic reviews
Source: PLOS Glob Public Health. 2025 May 7;5(5):e0004575. doi: 10.1371/journal.pgph.0004575 (PMC12057917; doi:10.1371/journal.pgph.0004575)
Supplement: S1 Appendix — (DOCX) [file pgph.0004575.s004.docx]

**S1 Appendix. Search Strategy**

**Ovid MEDLINE(R)**

1 adrenal cortex hormones/ or glucocorticoids/ or betamethasone/ or dexamethasone/

2 (glucocorticoid* or betamethasone or dexamethasone or corticosteroid* or steroid*).mp.

3 (decaject or decameth or dexasone or dexpak or hexadecadrol or hexadrol or maxidex or methylfluorprednisolone or millicorten or oradexon).mp.

4 (betadexamethasone or celeston or celestona or celestone or cellestoderm or flubenisolone).mp.

5 1 or 2 or 3 or 4

6 obstetric labor, premature/ or premature birth/

7 infant, premature/ or infant, extremely premature/

8 Term Birth/

9 Prenatal Care/

10 ((prenatal or antenatal or pre-natal or ante-natal) adj care).mp.

11 Prenatal Exposure Delayed Effects/

12 ((preterm or prematur* or term or fullterm) adj2 (infant* or birth* or child* or deliver* or labour or labor or born)).mp.

13 6 or 7 or 8 or 9 or 10 or 11 or 12

14 5 and 13

15 ((prenatal or antenatal or pre-natal or ante-natal) adj4 (glucocorticoid* or betamethasone or dexamethasone or corticosteroid* or steroid*)).mp.

16 (gestational age adj4 (glucocorticoid* or betamethasone or dexamethasone or corticosteroid* or steroid*)).mp.

17 ((("26" or "27" or "28" or "29" or "30" or "31" or "32" or "33" or "34" or "35" or "36") adj weeks adj6 (glucocorticoid* or betamethasone or dexamethasone or corticosteroid* or steroid*)) and (gestation* or pregnan* or trimester* or prenatal or antenatal or pre-natal or ante-natal)).mp. [mp=title, book title, abstract, original title, name of substance word, subject heading word, floating sub-heading word, keyword heading word, organism supplementary concept word, protocol supplementary concept word, rare disease supplementary concept word, unique identifier, synonyms, population supplementary concept word, anatomy supplementary concept word]

18 15 or 16 or 17

19 14 or 18

20 meta-analysis/ or "systematic review"/

21 "review"/

22 (meta-analy* or metaanaly* or metanalys#s).mp,pt.

23 (systematic* adj5 (review* or overview*)).mp,pt.

24 review.ti,pt.

25 (quantitative* adj5 (review* or overview* or synthes#s)).mp.

26 (methodologic* adj5 (review* or overview*)).mp.

27 (integrative research review* or research integration).mp.

28 20 or 21 or 22 or 23 or 24 or 25 or 26 or 27

**Embase Classic+Embase**

1 corticosteroid therapy/ or corticosteroid/

2 glucocorticoid/

3 betamethasone acetate/ or betamethasone/ or betamethasone sodium phosphate/ or betamethasone acetate plus betamethasone sodium phosphate/

4 dexamethasone/ or dexamethasone sodium phosphate/

5 (glucocorticoid* or betamethasone or dexamethasone or corticosteroid* or steroid*).mp.

6 (decaject or decameth or dexasone or dexpak or hexadecadrol or hexadrol or maxidex or methylfluorprednisolone or millicorten or oradexon).mp.

7 (betadexamethasone or celeston or celestona or celestone or cellestoderm or flubenisolone).mp.

8 premature labor/ or "immature and premature labor"/

9 prematurity/

10 term birth/

11 prenatal care/

12 prenatal exposure/ or prenatal drug exposure/

13 ((preterm or prematur* or term or fullterm) adj2 (infant* or birth* or child* or deliver* or labour or labor or born)).mp.

14 1 or 2 or 3 or 4 or 5 or 6 or 7

15 8 or 9 or 10 or 11 or 12 or 13

16 14 and 15

17 ((prenatal or antenatal or pre-natal or ante-natal) adj4 (glucocorticoid* or betamethasone or dexamethasone or corticosteroid* or steroid*)).mp.

18 (gestational age adj4 (glucocorticoid* or betamethasone or dexamethasone or corticosteroid* or steroid*)).mp.

19 ((("26" or "27" or "28" or "29" or "30" or "31" or "32" or "33" or "34" or "35" or "36") adj weeks adj6 (glucocorticoid* or betamethasone or dexamethasone or corticosteroid* or steroid*)) and (gestation* or pregnan* or trimester* or prenatal or antenatal or pre-natal or ante-natal)).mp. [mp=title, abstract, heading word, drug trade name, original title, device manufacturer, drug manufacturer, device trade name, keyword heading word, floating subheading word, candidate term word]

20 17 or 18 or 19

21 16 or 20

22 "systematic review"/ or "review"/

23 meta analysis/ or network meta-analysis/

24 (meta-analy* or metaanaly* or metanalys#s).mp,pt.

25 (systematic* adj5 (review* or overview*)).mp,pt.

26 review.ti,pt.

27 (quantitative* adj5 (review* or overview* or synthes#s)).mp.

28 (methodologic* adj5 (review* or overview*)).mp.

29 (integrative research review* or research integration).mp.

30 22 or 23 or 24 or 25 or 26 or 27 or 28 or 29

31 21 and 30

**Web of Science (inception to 2024)**

1: TS=(glucocorticosteroid*)

2: TS=(betamethasone)

3: TS=(dexamethasone )

4: ALL=(betadexamethasone or celeston or celestona or celestone or cellestoderm or flubenisolone)

5: TS=(decaject or decameth or dexasone or dexpak or hexadecadrol or hexadrol or maxidex or methylfluorprednisolone or millicorten or oradexon)

6: TS=((prenatal or antenatal or pre-natal or ante-natal) NEAR/2 care)

7: TS=((preterm or prematur* or term or fullterm) NEAR/2 (infant* or birth* or child* or deliver* or labour or labor or born))

8: #1 OR #2 OR #3 OR #4 OR #5

9: #6 OR #7

10: #8 AND #9

11: TS=((prenatal or antenatal or pre-natal or ante-natal) NEAR/4 (glucocorticoid* or betamethasone or dexamethasone or corticosteroid* or steroid*))

12: TS=(gestational age NEAR/4 (glucocorticoid* or betamethasone or dexamethasone or corticosteroid* or steroid*))

13: TS=(("26" or "27" or "28" or "29" or "30" or "31" or "32" or "33" or "34" or "35" or "36") NEAR weeks NEAR/6 (glucocorticoid* or betamethasone or dexamethasone or corticosteroid* or steroid*) and (gestation* or pregnan* or trimester* or prenatal or antenatal or pre-natal or ante-natal))

14: #11 OR #12 OR #13

15: #10 OR #14

16: TS=(systematic review)

17: DT=(Review)

18: TS=(meta-analy* or metaanaly*)

19: TS=(quantitative* NEAR/5 (review* or overview* or systhes*))

20: TS=(methodologic* NEAR/5 (review* or overview*))

21: TS=(integrative research review* or research integration)

22: #16 OR #17 OR #18 OR #19 OR #20 OR #21

23: #15 AND #22

**CINAHL**

S1 MH adrenal cortex hormones

S2 MH glucocorticoids

S3 MH betamethasone

S4 MH dexamethasone

S5 glucocorticoid* or betamethasone or dexamethasone or corticosteroid* or steroid*

S6 betadexamethasone or celeston or celestona or celestone or cellestoderm or flubenisolon

S7 decameth or dexasone or dexpak or hexadecadrol or hexadrol or maxidex or methylfluorprednisolone or millicorten or oradexon

S8 S1 OR S2 OR S3 OR S4 OR S5 OR S6 OR S7

S9 (MH "Infant, Premature")

S10 (MH "Childbirth, Premature")

S11 (MH "Labor, Premature")

S12 (MH "Term Birth")

S13 (MH "Prenatal Care")

S14 (MH "Prenatal Exposure Delayed Effects")

S15 ((preterm or prematur* or term or fullterm) N2 (infant* or birth* or child* or deliver* or labour or labor or born))

S16 S9 OR S10 OR S11 OR S12 OR S13 OR S14 OR

S17 S8 AND S16

S18 ((prenatal or antenatal or pre-natal or ante-natal) N4 (glucocorticoid* or betamethasone or dexamethasone or corticosteroid* or steroid*))

S19 (gestational age N4 (glucocorticoid* or betamethasone or dexamethasone or corticosteroid* or steroid*))

S20 (("26" or "27" or "28" or "29" or "30" or "31" or "32" or "33" or "33" or "34" or "35" or "36") N weeks N6 (glucocorticoid* or betamethasone or dexamethasone or corticosteroid* or steroid*)) and (gestation* or pregnan* or trimester* or prenatal or antenatal or pre-natal or ante- natal)) 3

S21 S18 OR S19 OR S20

S22 S17 OR S21

S23 (MH "Systematic Review") OR (MH "Scoping Review")

S24 (MH "Meta Analysis")

S25 meta-analy* or metaanaly* or metanalys#s

S26 (systematic* N5 (review* or overview*))

S27 PT review

S28 (quantitative* N5 (review* or overview* or synthes#s))

S29 TI review

S30 (methodologic* N5 (review* or overview*))

S31 (integrative research review* or research integration)

S32 S23 OR S24 OR S25 OR S26 OR S27 OR S28 OR S29 OR S30 OR S31

**Cochrane library**

#1 MeSH descriptor: [Adrenal Cortex Hormones] this term only

#2 MeSH descriptor: [Glucocorticoids] this term only

#3 MeSH descriptor: [Dexamethasone] this term only

#4 MeSH descriptor: [Betamethasone] this term only

#5 (decaject or decameth or dexasone or dexpak or hexadecadrol or hexadrol or maxidex or methylfluorprednisolone or millicorten or oradexon):ti,ab,kw

#6 (betadexamethasone or celeston or celestona or celestone or cellestoderm or flubenisolone):ti,ab,kw

#7 #1 OR #2 OR #3 OR #4 OR #5 OR #6

#8 MeSH descriptor: [Premature Birth] this term only

#9 MeSH descriptor: [Obstetric Labor, Premature] this term only

#10 MeSH descriptor: [Prenatal Care] this term only

#11 ((preterm or prematur* or term or fullterm) NEAR/2 (infant* or birth* or child* or deliver* or labour or labor or born)):ti,ab,kw (Word variations have been searched)

#12 #8 OR #9 OR #10 OR #11

#13 #7 AND #12

#14 ((prenatal or antenatal or pre-natal or ante-natal) NEAR/4 (glucocorticoid* or betamethasone or dexamethasone or corticosteroid* or steroid*)):ti,ab,kw

#15 ((gestational age) NEAR/4 (glucocorticoid* or betamethasone or dexamethasone or corticosteroid* or steroid*)):ti,ab,kw

#16 (((("26" or "27" or "28" or "29" or "30" or "31" or "32") NEAR weeks NEAR/6 (glucocorticoid* or betamethasone or dexamethasone or corticosteroid* or steroid*)) AND (gestation* or pregnan* or trimester* or prenatal or antenatal or pre-natal or ante-natal))):ti,ab,kw

#17 #14 OR #15 OR #16

#18 #13 OR #17

#19 #13 OR #17 in Cochrane Reviews, Cochrane Protocols

**Epistemonikos**

(antenatal OR prenatal) AND (corticosteroid* OR glucocorticoid* OR betamethasone OR dexamethaso ne OR steroid*) Systematic reviews
