# Supplementary material for: Long-term outcomes of antenatal corticosteroids for preterm birth: An overview of systematic reviews
Source: PLOS Glob Public Health. 2025 May 7;5(5):e0004575. doi: 10.1371/journal.pgph.0004575 (PMC12057917; doi:10.1371/journal.pgph.0004575)
Supplement: S3 Appendix — (DOCX) [file pgph.0004575.s006.docx]

**S3 Appendix. List of excluded studies with reason(s) for exclusion**

| **Study ID** | **Title** | **Authors** | **Reason of exclusion** |
| --- | --- | --- | --- |
| Zhu 2023 | The role of antenatal corticosteroids in twin pregnancy | Zhu J.; Li S.; Zhao Y.; Xiong Y. | wrong study design (not a systematic review) |
| Socha 2022 | Antenatal Corticosteroids and Neonatal Outcomes in Twins: A Systematic Review and Meta-analysis | Socha, Peter; McGee, Alice; Bhattacharya, Sohinee; Young, Catriona; Wang, Rui | no long-term outcomes reported |
| Patil 2022 | Antenatal dexamethasone reduces mortality in preterm infants in low-resource countries | Patil, Monika S; Gandhi, Bheru B; Gowda, Sharada H | wrong study design (not a systematic review) |
| Shinwell 2022 | Current evidence for prenatal and postnatal corticosteroids in preterm infants | Shinwell, Eric S; Gurevitz, Polina; Portnov, Igor | wrong study design (not a systematic review) |
| Uggioni 2022 | Corticosteroids in Pregnancy for Preventing RDS: Overview of Systematic Reviews | Uggioni, Maria Laura Rodrigues; Colonetti, Tamy; Grande, Antonio Jose; Cruz, Mateus Vinicius Barbosa; da Rosa, Maria Ines | no long-term outcomes reported |
| Asztalos 2022 | A Growing Dilemma: Antenatal Corticosteroids and Long-Term Consequences | Asztalos, Elizabeth V; Murphy, Kellie E; Matthews, Stephen G | wrong study design (not a systematic review) |
| Liauw 2022 | Technical Update No. 439: Antenatal Corticosteroids at Late Preterm Gestation | Liauw J.; Foggin H.; Socha P.; Crane J.; John's S.; Joseph K.S.; Burrows J.; Lacaze-Masmonteil T.; Jain V.; Boutin A.; Hutcheon J. | wrong study design (not a systematic review) |
| Zeng 2022 | Beyond Fetal Immunity: A Systematic Review and Meta-Analysis of the Association Between Antenatal Corticosteroids and Retinopathy of Prematurity | Zeng Y.; Ge G.; Lei C.; Zhang M. | no long-term outcomes reported |
| Walters 2022 | Repeat prenatal corticosteroids for women at risk of preterm birth for improving neonatal health outcomes | Walters A.G.B.; McKinlay C.J.D.; Middleton P.; Harding J.E.; Crowther C.A. | conference abstract |
| Samouilidis 2022 | The Use of Antenatal Dexamethasone in Late Preterm and Term Pregnancies to Improve Neonatal Morbidity and Mortality: A Systematic Review and Meta-Analysis | Samouilidis, A; Beltsios, ET; Mavrovounis, G; Adamou, A; Belios, I; Hadjivasilis, A; Pantazopoulos, I; Agouridis, AP | no long-term outcomes reported |
| Lin 2021 | Association of antenatal corticosteroids with morbidity and mortality among preterm multiple gestations: meta-analysis of observational studies | Lin, Dongxin; Fan, Dazhi; Chen, Gengdong; Luo, Caihong; Guo, Xiaoling; Liu, Zhengping | no long-term outcomes reported |
| Kleinhout 2021 | Evidence-based interventions to reduce mortality among preterm and low-birthweight neonates in low-income and middle-income countries: a systematic review and meta-analysis | Kleinhout, Mirjam Y; Stevens, Merel M; Osman, Kwabena Aqyapong; Adu-Bonsaffoh, Kwame; Groenendaal, Floris; Biza Zepro, Nejimu; Rijken, Marcus J; Browne, Joyce L | no long-term outcomes reported |
| Mwita 2021 | Reducing neonatal mortality and respiratory distress syndrome associated with preterm birth: a scoping review on the impact of antenatal corticosteroids in low- and middle-income countries | Mwita, Stanley; Jande, Mary; Katabalo, Deogratias; Kamala, Benjamin; Dewey, Deborah | no long-term outcomes reported |
| Ninan 2021 | Neonatal and Maternal Outcomes of Lower Versus Standard Doses of Antenatal Corticosteroids for Women at Risk of Preterm Delivery: A Systematic Review of Randomized Controlled Trials | Ninan, Kiran; Morfaw, Frederick; Murphy, Kellie E; Beyene, Joseph; McDonald, Sarah D | no long-term outcomes reported |
| Busuulwa 2021 | The role of antenatal corticosteroids in improving neonatal outcomes | Busuulwa P.; Groom K.; Chappell L.C.; Shennan A.H. | wrong study design (not a systematic review) |
| Ninan 2021 | Long-term neurodevelopmental and psychological outcomes after prenatal exposure to antenatal corticosteroids: a systematic review and meta-analysis | Ninan K.; Liyanage S.; Murphy K.; Asztalos E.; McDonald S. | conference abstract |
| Nag 2021 | Does the risk of antenatal corticosteroids outweigh benefits in neonates born to diabetic mothers delivering in late pre-term or term? | Nag G. | conference abstract |
| Deshmukh 2021 | Antenatal corticosteroids for impending late preterm (34-36+6 weeks) deliveries-A systematic review and meta-analysis of RCTs | Deshmukh, Mangesh; Patole, Sanjay | no long-term outcomes reported |
| Rohwer 2020 | Strategies for optimising antenatal corticosteroid administration for women with anticipated preterm birth | Rohwer, Anke C; Oladapo, Olufemi T; Hofmeyr, G Justus | wrong intervention (corticosteroids not administered for preterm birth) |
| Novak 2020 | State of the Evidence Traffic Lights 2019: Systematic Review of Interventions for Preventing and Treating Children with Cerebral Palsy | Novak, Iona; Morgan, Catherine; Fahey, Michael; Finch-Edmondson, Megan; Galea, Claire; Hines, Ashleigh; Langdon, Katherine; Namara, Maria Mc; Paton, Madison Cb; Popat, Himanshu; Shore, Benjamin; Khamis, Amanda; Stanton, Emma; Finemore, Olivia P; Tricks, Alice; Te Velde, Anna; Dark, Leigha; Morton, Natalie; Badawi, Nadia | wrong study design (not a systematic review) |
| Backes 2020 | Proactive Neonatal Treatment at 22 Weeks of Gestation: A Systematic Review and Meta-Analysis | Backes C.H.; Rivera B.K.; Pavlek L.; Beer L.J.; Ball M.K.; Zettler E.T.; Smith C.V.; Bridge J.A.; Bell E.F.; Frey H.A. | wrong intervention (corticosteroids not administered for preterm birth) |
| Gibbons 2020 | Predicting Lung Health Trajectories for Survivors of Preterm Birth | Gibbons J.T.D.; Wilson A.C.; Simpson S.J. | wrong study design (not a systematic review) |
| Karnati 2020 | Late preterm infants - Changing trends and continuing challenges | Karnati S.; Kollikonda S.; Abu-Shaweesh J. | wrong study design (not a systematic review) |
| Deshmukh 2020 | Antenatal corticosteroids for impending late preterm (34-36+6 weeks) deliveries-current evidence from RCTS | Deshmukh M.; Patole S. | conference abstract |
| Gubert 2020 | Rescue steroids after administration remote from delivery: A systematic review of the literature | Gubert P.; Murphy K.E.; Ryu M.; Ladhani N.N.N. | conference abstract |
| Pofi 2020 | Glucocorticoids in pregnancy | Pofi, R; Tomlinson, JW | wrong study design (not a systematic review) |
| Chen 2019 | Prenatal glucocorticoids exposure and fetal adrenal developmental programming | Chen, Yawen; He, Zheng; Chen, Guanghui; Liu, Min; Wang, Hui | wrong study design (not a systematic review) |
| Groom 2019 | Antenatal corticosteroids after 34weeks' gestation: Do we have the evidence? | Groom, Katie M | wrong study design (not a systematic review) |
| Jobe 2019 | Neonatal stress and resilience - lasting effects of antenatal corticosteroids 1 | Jobe, Alan H | wrong study design (not a systematic review) |
| Franks 2019 | Prenatal Drug Exposures and Neurodevelopmental Programming of Glucocorticoid Signaling | Franks A.L.; Berry K.J.; DeFranco D.B. | wrong study design (not a systematic review) |
| Kumagai 2019 | Contemporary Challenges and Developments: Antenatal Corticosteroid Therapy | Kumagai Y.; Kemp M.W.; Yaegashi N.; Saito M. | wrong study design (not a systematic review) |
| Khodzhaeva 2019 | Antenatal prevention of fetal respiratory distress syndrome: A glimpse into the future | Khodzhaeva Z.S.; Gorina K.A. | wrong study design (not a systematic review) |
| Cherak 2019 | Prenatal glucocorticoids and child neuroimaging: A systematic review | Cherak S.J.; Bahador R.; Paniukov D.; Lopez G.C.; Williamson T.; Lebel C.; Giesbrecht G.F. | conference abstract |
| Young 2019 | Efficacy of antenatal corticosteroids for preventing neonatal respiratory distress syndrome in twin pregnancies at risk of preterm birth: Systematic review and meta-analysis of randomised and observational data | Young C.; Bhattacharya S. | conference abstract |
| Blankenship 2019 | 483: Antenatal corticosteroids in preterm growth-restricted fetuses: A systematic review and meta- analysis | Blankenship S.A.; Brown K.E.; Stout M.J.; Tuuli M.G. | conference abstract |
| Jobe 2019 | Neonatal stress and resilience - lasting effects of antenatal corticosteroids | Jobe, AH | wrong study design (not a systematic review) |
| Skoll 2018 | No. 364-Antenatal Corticosteroid Therapy for Improving Neonatal Outcomes | Skoll, Amanda; Boutin, Amelie; Bujold, Emmanuel; Burrows, Jason; Crane, Joan; Geary, Michael; Jain, Venu; Lacaze-Masmonteil, Thierry; Liauw, Jessica; Mundle, William; Murphy, Kellie; Wong, Suzanne; Joseph, K S | wrong study design (not a systematic review) |
| Dresang 2018 | Clinical Inquiries: What are the benefits/risks of giving betamethasone to women at risk of late preterm labor? | Dresang, Lee; Hooper-Lane, Christopher | wrong study design (not a systematic review) |
| Dixon 2018 | Past and Present: A Review of Antenatal Corticosteroids and Recommendations for Late Preterm Birth Steroids | Dixon, C Luke; Too, Gloria; Saade, George R; Gyamfi-Bannerman, Cynthia | wrong study design (not a systematic review); |
| Twilhaar 2018 | Cognitive Outcomes of Children Born Extremely or Very Preterm Since the 1990s and Associated Risk Factors: A Meta-analysis and Meta-regression | Twilhaar, E Sabrina; Wade, Rebecca M; de Kieviet, Jorrit F; van Goudoever, Johannes B; van Elburg, Ruurd M; Oosterlaan, Jaap | wrong intervention (corticosteroids not administered for preterm birth) |
| Deshmukh 2018 | Antenatal corticosteroids in impending preterm deliveries before 25 weeks' gestation | Deshmukh, Mangesh; Patole, Sanjay | no long-term outcomes reported) |
| Twilhaar 2018 | Academic performance of children born preterm: a meta-analysis and meta-regression | Twilhaar, E Sabrina; de Kieviet, Jorrit F; Aarnoudse-Moens, Cornelieke Sh; van Elburg, Ruurd M; Oosterlaan, Jaap | wrong intervention |
| Twilhaar 2018 | Cognitive Outcomes of Children Born Extremely or Very Preterm since the 1990s and Associated Risk Factors: A Meta-analysis and Meta-regression | Twilhaar E.S.; Wade R.M.; De Kieviet J.F.; Van Goudoever J.B.; Van Elburg R.M.; Oosterlaan J. | wrong study design (not a systematic review) |
| Rodriguez-Bosch 2018 | Management of premature preterm rupture of membranes (24-33.6 weeks): Recent scientific evidence | Rodriguez-Bosch M.R.; Miranda-Araujo O.; Resendiz-Rossetti A.E. | wrong study design (not a systematic review) |
| Ndege 2018 | Mental health outcomes in offspring exposed to antenatal corticosteroids for threatened preterm labor: a systematic review of the literature | Ndege E.B.C.; Ayorinde A.; Bhattacharya S. | conference abstract |
| Osman 2018 | Interventions to reduce premature births: a review of the evidence | Osman R.; Manikam L.; Watters K. | conference abstract |
| Thiele 2018 | Antenatal corticosteroids in preterm small-for-gestational-age infants: What is the evidence? | Thiele C. | wrong study design (not a systematic review) |
| Kugelman 2018 | Antenatal and Postnatal Corticosteroids for preterm infants | Kugelman A. | conference abstract |
| Deshmukh 2018 | Antenatal corticosteroids in impending preterm deliveries before 25 weeks gestation: A systematic review and metaanalysis | Deshmukh M.; Patole S. | conference abstract |
| Murray 2017 | Long-term childhood outcomes after interventions for prevention and management of preterm birth | Murray, Sarah R; Stock, Sarah J; Norman, Jane E | wrong study design (not a systematic review); |
| Shepherd 2017 | Antenatal and intrapartum interventions for preventing cerebral palsy: an overview of Cochrane systematic reviews | Shepherd, Emily; Salam, Rehana A; Middleton, Philippa; Makrides, Maria; McIntyre, Sarah; Badawi, Nadia; Crowther, Caroline A | wrong study design (not a systematic review) |
| Vaivada 2017 | Promoting Early Child Development With Interventions in Health and Nutrition: A Systematic Review | Vaivada, Tyler; Gaffey, Michelle F; Bhutta, Zulfiqar A | wrong study design (not a systematic review) |
| Travers 2017 | Antenatal corticosteroid administration between 24 hours and 7 days before extremely preterm delivery is associated with the lowest rate of mortality | Travers, Colm P; Carlo, Waldemar A | wrong study design (not a systematic review) |
| Deshmukh 2017 | Antenatal corticosteroids for neonates born before 25 Weeks-A systematic review and meta-analysis | Deshmukh, Mangesh; Patole, Sanjay | no long- term outcomes reported |
| Roberts 2017 | Antenatal corticosteroids for accelerating fetal lung maturation for women at risk of preterm birth | Roberts, Devender; Brown, Julie; Medley, Nancy; Dalziel, Stuart R | previous version of an updated review |
| Shaughnessy 2017 | Steroids at 34 to 36 Weeks' and Before Term Cesarean Decrease Respiratory Distress Syndrome | Shaughnessy, Allen F | wrong study design (not a systematic review) |
| Magann 2017 | Use of antenatal corticosteroids in special circumstances: a comprehensive review | Magann, Everett F; Haram, Kjell; Ounpraseuth, Songthip; Mortensen, Jan H; Spencer, Horace J; Morrison, John C | no long-term outcomes reported |
| Roberts 2017 | Antenatal corticosteroids for accelerating fetal lung maturation for women at risk of preterm birth | Roberts D.; Brown J.; Medley N.; Dalziel S.R. | previous version of an updated review |
| Rose 2017 | Optimizing Care and Outcomes for Late Preterm Neonates | Rose R.; Engle W.A. | wrong study design (not a systematic review) |
| Saccone 2016 | Antenatal corticosteroids for maturity of term or near term fetuses: systematic review and meta-analysis of randomized controlled trials | Saccone, Gabriele; Berghella, Vincenzo | no long-term outcomes reported |
| Solano 2016 | Antenatal endogenous and exogenous glucocorticoids and their impact on immune ontogeny and long-term immunity | Solano, Maria Emilia; Holmes, Megan C; Mittelstadt, Paul R; Chapman, Karen E; Tolosa, Eva | wrong study design (not a systematic review) |
| Kamath-Rayne 2016 | Antenatal corticosteroids beyond 34 weeks gestation: What do we do now? | Kamath-Rayne, Beena D; Rozance, Paul J; Goldenberg, Robert L; Jobe, Alan H | wrong study design (not a systematic review) |
| Cassiano 2016 | Prematurity, neonatal health status, and later child behavioral/emotional problems: a systematic review | Cassiano, Rafaela G M; Gaspardo, Claudia M; Linhares, Maria Beatriz M | wrong intervention |
| Bensley 2016 | The effects of preterm birth and its antecedents on the cardiovascular system | Bensley, Jonathan G; De Matteo, Robert; Harding, Richard; Black, Mary J | wrong study design (not a systematic review) |
| Nijman 2016 | Antepartum and intrapartum interventions to prevent preterm birth and its sequelae | Nijman, T A J; van Vliet, E O G; Koullali, B; Mol, B W; Oudijk, M A | wrong study design (not a systematic review) |
| Linsell 2016 | Prognostic factors for cerebral palsy and motor impairment in children born very preterm or very low birthweight: a systematic review | Linsell, Louise; Malouf, Reem; Morris, Joan; Kurinczuk, Jennifer J; Marlow, Neil | wrong intervention (corticosteroids not administered for preterm birth) |
| Constantinof 2016 | Programming of stress pathways: A transgenerational perspective | Constantinof, Andrea; Moisiadis, Vasilis G; Matthews, Stephen G | wrong study design (not a systematic review) |
| Twilhaar 2016 | Academic performance of preterm children born in the antenatal steroids and surfactant era: A meta-analysis | Twilhaar E.S.; De Kieviet J.F.; Aarnoudse-Moens C.S.H.; Van Elburg R.M.; Oosterlaan J. | conference abstract |
| Cartier 2016 | Glucocorticoids and the prenatal programming of neurodevelopmental disorders | Cartier J.; Zeng Y.; Drake A.J. | wrong study design (not a systematic review) |
| Maben-Feaster 2016 | Antenatal Corticosteroid Therapy Before 24 Weeks of Gestation: A Systematic Review and Meta-analysis | Maben-Feaster, R; Truong, M; McHugh, KW; Chescheir, NC | worng study design |
| Saccone 2016 | Antenatal corticosteroids for maturity of term or near term fetuses: systematic review and meta-analysis of randomised controlled trials | Saccone, G | wrong study design (not a systematic review) |
| Segerer 2016 | Lung Function in Childhood and Adolescence: Influence of Prematurity and Bronchopulmonary Dysplasia | Segerer, FJH; Speer, CP | wrong intervention |
| Crowther 2015 | Repeat doses of prenatal corticosteroids for women at risk of preterm birth for improving neonatal health outcomes | Crowther, Caroline A; McKinlay, Christopher J D; Middleton, Philippa; Harding, Jane E | previous version of an updated review |
| Msan 2015 | Use of antenatal corticosteroids in the management of preterm delivery | Msan, Anthony K; Usta, Ihab M; Mirza, Fadi G; Nassar, Anwar H | wrong study design (not a systematic review) |
| Jarjour 2015 | Neurodevelopmental outcome after extreme prematurity: A review of the literature | Jarjour I.T. | no intervention |
| Brown 2015 | Should we give antenatal corticosteroids to women with diabetes in pregnancy at term for fetal lung maturation? | Brown J.A.; Alsweiler J.; Crawford T.; McGoldrick E.; Middleton P.F.; Crowther C.A. | wrong intervention |
| Ireland 2015 | Factors influencing the care provided for periviable babies in Australia: a narrative review | Ireland, Susan; Ray, Robin; Larkins, Sarah; Woodward, Lynn | no intervention |
| Gore 2014 | Implications of prenatal steroid perturbations for neurodevelopment, behavior, and autism | Gore, Andrea C; Martien, Katherine M; Gagnidze, Khatuna; Pfaff, Donald | wrong study design (not a systematic review) |
| MachadoJunior 2014 | Late prematurity: a systematic review | Machado Junior, Luis Carlos; Passini Junior, Renato; Rodrigues Machado Rosa, Izilda | no long-term outcomes reported |
| Zhang 2014 | Effect of dexamethasone on intelligence and hearing in preterm infants: A meta-analysis | Zhang R.L.; Bo T.; Shen L.; Luo S.L.; Li J. | wrong intervention (corticosteroids not administered for preterm birth) |
| Chang 2014 | Evidence for adverse effect of perinatal glucocorticoid use on the developing brain | Chang Y.P. | wrong study design (not a systematic review) |
| Crowther 2014 | Antenatal corticosteroids to reduce preterm deaths in low-income settings | Crowther C.; Brown J. | wrong study design (not a systematic review) |
| Fuchs 2014 | Prenatal corticosteroids: Short-term and long-term effects of multiple courses. A literature review | Fuchs F.; Audibert F.; Senat M.-V. | wrong study design (not a systematic review) |
| Manojlovic-Stojanoski 2014 | Antenatal glucocorticoid therapy and the hypothalamic-pituitary-adrenal axis | Manojlovic-Stojanoski, M; Ristic, N; Singh, S; Milosevic, V | wrong study design (not a systematic review) |
| Wapner 2013 | Antenatal corticosteroids for periviable birth | Wapner, Ronald J | wrong study design (not a systematic review) |
| Crowther 2013 | Thyrotropin-releasing hormone added to corticosteroids for women at risk of preterm birth for preventing neonatal respiratory disease | Crowther C.A.; Alfirevic Z.; Han S.; Haslam R.R. | wrong intervention |
| Brownfoot 2013 | Different corticosteroids and regimens for accelerating fetal lung maturation for women at risk of preterm birth | Brownfoot F.C.; Gagliardi D.I.; Bain E.; Middleton P.; Crowther C.A. | previous version of an updated review |
| Bos 2013 | Development of fine motor skills in preterm infants | Bos, AF; Van Braeckel, KNJA; Hitzert, MM; Tanis, JC; Roze, E | no intervention |
| Painter 2012 | Long-term effects of prenatal stress and glucocorticoid exposure | Painter, Rebecca C; Roseboom, Tessa J; de Rooij, Susanne R | wrong study design (not a systematic review) |
| McKinlay 2012 | Repeat antenatal glucocorticoids for women at risk of preterm birth: a Cochrane Systematic Review | McKinlay, Christopher J D; Crowther, Caroline A; Middleton, Philippa; Harding, Jane E | previous version of an updated review |
| Contopoulos-Ioannidis 2012 | Claims for improved survival from systemic corticosteroids in diverse conditions: an umbrella review | Contopoulos-Ioannidis, Despina G; Ioannidis, John P A | no long-term outcomes reported |
| Keunen 2012 | Brain tissue volumes in preterm infants: Prematurity, perinatal risk factors and neurodevelopmental outcome: A systematic review | Keunen K.; Kersbergen K.J.; Groenendaal F.; Isgum I.; De Vries L.S.; Benders M.J.N.L. | wrong intervention |
| Asztalos 2012 | Antenatal Corticosteroids: A Risk Factor for the Development of Chronic Disease | Asztalos, E | wrong study design (not a systematic review) |
| Utama 2011 | Transplacental versus direct fetal corticosteroid treatment for accelerating fetal lung maturation where there is a risk of preterm birth | Utama, Debby P; Crowther, Caroline A | no long-term outcomes reported |
| Crowther 2011 | Repeat doses of prenatal corticosteroids for women at risk of preterm birth for improving neonatal health outcomes | Crowther, Caroline A; McKinlay, Christopher Jd; Middleton, Philippa; Harding, Jane E | previous version of an updated review |
| Vidaeff 2011 | Antenatal corticosteroids after preterm premature rupture of membranes | Vidaeff, Alex C; Ramin, Susan M | wrong study design (not a systematic review |
| Roberge 2011 | Role of fetal sex in the outcome of antenatal glucocorticoid treatment to prevent respiratory distress syndrome: systematic review and meta-analysis | Roberge, Stephanie; Lacasse, Yves; Tapp, Sylvie; Tremblay, Yves; Kari, Anneli; Liu, Jing; Fekih, Myriam; Qublan, Hussein S; Amorim, Melania M; Bujold, Emmanuel | no long-term outcomes reported |
| Marciniak 2011 | Glucocorticoids in pregnancy | Marciniak, Beata; Patro-Malysza, Jolanta; Poniedzialek-Czajkowska, Elzbieta; Kimber-Trojnar, Zaneta; Leszczynska-Gorzelak, Bozena; Oleszczuk, Jan | wrong study design (not a systematic review) |
| Harris 2011 | Glucocorticoids, prenatal stress and the programming of disease | Harris, Anjanette; Seckl, Jonathan | wrong study design (not a systematic review) |
| Crowther 2011 | Repeat dose prenatal corticosteroids for women at risk of preterm birth: The cochrane review | Crowther C.A.; McKinlay C.J.D.; Middleton P.; Harding J.E. | conference abstract |
| Bevilacqua 2010 | Review and meta-analysis: Benefits and risks of multiple courses of antenatal corticosteroids | Bevilacqua, Elisa; Brunelli, Roberto; Anceschi, Maurizio M | no long-term outcomes reported |
| McKinlay 2010 | Repeat doses of antenatal glucocorticoids for women at risk of preterm birth: An updated systematic review | McKinlay C.J.; Harding J.E.; Crowther C.A. | conference abstract |
| Anceschi 2010 | Antenatal steroids: Are we confident on the evidence | Anceschi M.M.; Bevilacqua E. | wrong study design (not a systematic review) |
| Shinwell 2009 | Impact of perinatal corticosteroids on neuromotor development and outcome: review of the literature and new meta-analysis | Shinwell, E S; Eventov-Friedman, S | wrong study design (not a systematic review) |
| Guilherme 2009 | Repeat doses of prenatal corticosteroids for women at risk of preterm birth: A difficult consensus | Guilherme R.; Renaud C.; Dommergues M.; Mitanchez D. | wrong study design (not a systematic review) |
| Brownfoot 2008 | Different corticosteroids and regimens for accelerating fetal lung maturation for women at risk of preterm birth | Brownfoot, Fiona C; Crowther, Caroline A; Middleton, Philippa | previous version of an updated review |
| Marret 2008 | [Is it possible to protect the preterm infant brain and to decrease later neurodevelopmental disabilities?]. | Marret, S; Foix-L'helias, L; Ancel, P-Y; Kaminski, M; Larroque, B; Marcou-Labarre, A; Laudenbach, V | wrong study design (not a systematic review) |
| Kapoor 2008 | Fetal programming of hypothalamic-pituitary-adrenal (HPA) axis function and behavior by synthetic glucocorticoids | Kapoor A.; Petropoulos S.; Matthews S.G. | wrong study design (not a systematic review) |
| Eventov-Friedman 2008 | Current controversies in perinatal steroid therapy | Eventov-Friedman S.; Shinwell E.S. | wrong study design (not a systematic review) |
| Pole 2008 | Antenatal steroid therapy and childhood asthma: Is there a possible link? | Pole, JD; Mustard, CA; To, T; Beyene, J; Allen, AC | wrong study design (not a systematic review) |
| Crowther 2007 | Repeat doses of prenatal corticosteroids for women at risk of preterm birth for preventing neonatal respiratory disease | Crowther, C A; Harding, J E | previous version of an updated review |
| Asztalos 2007 | The need to go beyond: evaluating antenatal corticosteroid trials with long-term outcomes | Asztalos, Elizabeth | wrong study design (not a systematic review) |
| deVries 2007 | Long-term effects of perinatal glucocorticoid treatment on the heart | de Vries W.B.; van Oosterhout M.F.M.; Bal M.P.; Baan J.; Heijnen C.J.; van Bel F. | wrong study design (not a systematic review) |
| Brownfoot 2007 | Different corticosteroids and regimens for accelerating fetal lung maturation for women at risk of preterm birth | Brownfoot F.; Crowther C.A.; Middleton P. | previous version of an updated review |
| Roberts 2006 | Antenatal corticosteroids for accelerating fetal lung maturation for women at risk of preterm birth | Roberts, D; Dalziel, S | previous version of an updated review |
| Roberts 2006 | Antenatal corticosteroids for accelerating fetal lung maturation for women at risk of preterm birth (Review) | Roberts D.; Dalziel S. | previous version of an updated review |
| Narang 2006 | Airway function measurements and the long-term follow-up of survivors of preterm birth with and without chronic lung disease | Narang I.; Baraldi E.; Silverman M.; Bush A. | wrong study design (not a systematic review) |
| Velisek 2005 | Prenatal corticosteroid impact on hippocampus: implications for postnatal outcomes | Velisek, Libor | wrong study design (not a systematic review) |
| Sloboda 2005 | Synthetic glucocorticoids: antenatal administration and long-term implications | Sloboda, D M; Challis, J R G; Moss, T J M; Newnham, J P | wrong study design (not a systematic review) |
| Kent 2005 | Antenatal steroids may reduce adverse neurological outcome following chorioamnionitis: Neurodevelopmental outcome and chorioamnionitis in premature infants | Kent A.; Lomas F.; Hurrion E.; Dahlstrom J.E. | wrong study design (not a systematic review) |
| Crowther 2004 | Thyrotropin-releasing hormone added to corticosteroids for women at risk of preterm birth for preventing neonatal respiratory disease. | Crowther, C A; Alfirevic, Z; Haslam, R R | previous version of an updated review |
| Halliday 2004 | Use of steroids in the perinatal period | Halliday H.L. | wrong study design (not a systematic review) |
| Vidaeff 2003 | Antenatal corticosteroids for fetal maturation in women at risk for preterm delivery | Vidaeff, Alex C; Doyle, Nora M; Gilstrap, Larry C 3rd | wrong study design (not a systematic review) |
| McLaughlin 2003 | Effects of a single course of corticosteroids given more than 7 days before birth: a systematic review | McLaughlin, Kristin J; Crowther, Caroline A; Walker, Natalie; Harding, Jane E | no long-term outcomes reported |
| Arad 2003 | Developmental assessment of prematurely born children exposed to antenatal corticosteroids | Arad, Ilan; Bromiker, Ruben | wrong study design (not a systematic review) |
| Crowther 2003 | Repeat doses of prenatal corticosteroids for women at risk of preterm birth for preventing neonatal respiratory disease | Crowther, C A; Harding, J | previous version of an updated review |
| Rajadurai 2003 | The use and abuse of steroids in perinatal medicine | Rajadurai, V S; Tan, K H | wrong study design (not a systematic review) |
| Leung 2003 | Repeated courses of antenatal corticosteroids: is it justified? | Leung, Tse N; Lam, Po M; Ng, Pak C; Lau, Tze K | wrong study design (not a systematic review) |
| Ramsey 2002 | Therapies administered to mothers at risk for preterm birth and neurodevelopmental outcome in their infants | Ramsey, Patrick S; Rouse, Dwight J | wrong study design (not a systematic review) |
| Senat 2002 | [Corticosteroid for fetal lung maturation: indication and treatment protocols]. | Senat, M V | conference abstract |
| Cambonie 2002 | [Respiratory growth in the premature infant: development to 2 years of age]. | Cambonie, G; Counil, F | wrong study design (not a systematic review) |
| Narayan 2002 | Steroids in perinatology | Narayan S.; Deorari A.K. | wrong study design (not a systematic review) |
| O'Shea 2001 | Perinatal glucocorticoid therapy and neurodevelopmental outcome: an epidemiologic perspective | O'Shea, T M; Doyle, L W | wrong study design (not a systematic review) |
| Perlman 2001 | Neurobehavioral deficits in premature graduates of intensive care--potential medical and neonatal environmental risk factors | Perlman, J M | wrong study design (not a systematic review) |
| Bakker 2001 | Neonatal glucocorticoids and the developing brain: short-term treatment with life-long consequences? | Bakker, J M; van Bel, F; Heijnen, C J | wrong study design (not a systematic review) |
| Walfisch 2001 | Multiple courses of antenatal steroids: risks and benefits | Walfisch, A; Hallak, M; Mazor, M | no long-term outcomes reported |
| Bolt 2001 | Glucocorticoids and lung development in the fetus and preterm infant | Bolt, R J; van Weissenbruch, M M; Lafeber, H N; Delemarre-van de Waal, H A | wrong study design (not a systematic review) |
| Joy 2001 | Single course versus multiple courses of antenatal corticosteroids: A meta-analysis | Joy, S; Sanchez-Ramos, L; Kaunitz, A | conference abstract |
| Mastrobattista 2000 | Therapeutic agents in preterm labor: steroids | Mastrobattista, J M | wrong study design (not a systematic review) |
| Crowley 2000 | Prophylactic corticosteroids for preterm birth | Crowley, P | previous version of an updated review |
| Matthews 2000 | Antenatal glucocorticoids and programming of the developing CNS | Matthews, S G | wrong study design (not a systematic review) |
| Hack 1999 | Outcomes of children of extremely low birthweight and gestational age in the 1990's | Hack, M; Fanaroff, A A | wrong study design (not a systematic review) |
| Gilstrap 1995 | Effect of corticosteroids for fetal maturation on perinatal outcomes | Gilstrap L.C.; Christensen R.; Clewell W.H.; D'Alton M.E.; Davidson Jr. E.C.; Escobedo M.B.; Gjerdingen D.K.; Goddard-Finegold J.; Goldenberg R.L.; Grimes D.A.; Hansen T.N.; Kauffman R.E.; Keeler E.B.; Oh W.; Susman E.J.; Vogel M.G.; Avery M.E.; Ballard P.L.; Ballard R.A. | wrong study design (not a systematic review) |
| Gourrier 1994 | [Pre- or neonatal corticotherapy: what are the long-term consequences?] | Gourrier, E; Leraillez, J; Wood, C; Mouchnino, G; Merbouche, S | wrong study design (not a systematic review) |
| Ohlsson 1989 | Treatments of preterm premature rupture of the membranes: a meta-analysis | Ohlsson, A | no long-term outcomes reported |
