# Supplementary material for: Long-term outcomes of antenatal corticosteroids for preterm birth: An overview of systematic reviews
Source: PLOS Glob Public Health. 2025 May 7;5(5):e0004575. doi: 10.1371/journal.pgph.0004575 (PMC12057917; doi:10.1371/journal.pgph.0004575)
Supplement: S4 Appendix — (DOCX) [file pgph.0004575.s007.docx]

**S4 Appendix. Citation matrix**

|  | Ninan 2022 | McGoldrick 2020 | Park 2016 | Sotiriadis 2015 | Onland 2011 | Crowley 1995 | Ninan 2023b | Walters 2022 | Crowther 2019 | Peltoniemi 2011 | Aghajafari 2001 | Williams 2022 | Ciapponi 2021 | Blankenship 2020 | Wang 2022 | Sacco 2022 | Sarid 2022 | Amiya 2016 | Ninan 2023a |
| --- | --- | --- | --- | --- | --- | --- | --- | --- | --- | --- | --- | --- | --- | --- | --- | --- | --- | --- | --- |
| Amorium 1999 |  | X |  |  |  |  |  |  |  |  |  |  |  |  |  |  |  |  |  |
| Collaborative 1981 |  | X |  | X |  | X |  |  |  |  |  |  |  |  |  |  |  |  |  |
| Kari 1994 |  | X |  | X | X |  |  |  |  |  |  |  |  |  |  |  |  |  |  |
| Liggins 1972 |  | X |  | X |  | X |  |  |  |  |  |  |  |  |  | X |  |  |  |
| Schutte 1980 |  | X |  | X |  | X |  |  |  |  |  |  |  |  |  |  |  |  |  |
| Argarwal 2018 | X |  |  |  |  |  |  |  |  |  |  |  |  |  |  |  |  |  |  |
| Aviram 2021 | X |  |  |  |  |  |  |  |  |  |  |  |  |  |  |  |  |  | X |
| Basset 2018 | X |  |  |  |  |  |  |  |  |  |  |  |  |  |  |  |  |  |  |
| Bulbul 2020 | X |  |  |  |  |  |  |  |  |  |  |  |  |  |  |  |  |  |  |
| Chawla 2013 | X |  |  | X |  |  |  |  |  |  |  |  |  |  | X |  |  |  |  |
| Chawla 2016 | X |  |  |  |  |  |  |  |  |  |  |  |  |  | X |  |  |  |  |
| Gentle 2020 | X |  |  |  |  |  |  |  |  |  |  |  |  |  |  |  |  |  |  |
| Gover 2012 | X |  |  |  |  |  |  |  |  |  |  |  |  |  |  |  |  |  |  |
| Haslam 2018 | X |  |  |  |  |  |  |  |  |  |  |  |  |  |  |  |  |  |  |
| Hutcheon 2020 | X |  |  |  |  |  |  |  |  |  |  |  |  |  |  |  |  |  |  |
| Ishikawa 2015 | X |  |  |  |  |  |  |  |  |  |  |  |  | X |  |  |  |  |  |
| Kallen 2015 | X |  |  |  |  |  |  |  |  |  |  |  |  |  |  |  |  |  |  |
| Kiechl-Kohlendorfer 2009 | X |  |  |  |  |  |  |  |  |  |  |  |  |  |  |  |  |  |  |
| Kim 2018 | X |  |  |  |  |  |  |  |  |  |  |  |  |  |  |  |  |  |  |
| Lamminmaki 2021 | X |  |  |  |  |  |  |  |  |  |  |  |  |  |  |  |  |  |  |
| Lardon 2017 | X |  |  |  |  |  |  |  |  |  |  |  |  |  |  |  |  |  |  |
| Laughon 2009 | X |  |  |  |  |  |  |  |  |  |  |  |  |  |  |  |  |  |  |
| Lee 2008 | X |  |  | X |  |  |  |  |  |  |  |  |  |  | X |  |  |  |  |
| Li 2019 | X |  |  |  |  |  |  |  |  |  |  |  |  |  |  |  |  |  |  |
| Melamed 2019 | X |  |  |  |  |  |  |  |  |  |  |  |  |  |  |  | X |  | X |
| McElrath 2009 | X |  |  |  |  |  |  |  |  |  |  |  |  |  |  |  |  |  |  |
| Miyazaki 2015 | X |  |  |  |  |  |  |  |  |  |  |  |  |  | X |  |  |  |  |
| Ochiai 2014 | X |  |  |  |  |  |  |  |  |  |  |  |  |  |  |  |  |  |  |
| Raikkonen 2020 | X |  |  |  |  |  |  |  |  |  |  |  |  |  |  |  | X |  | X |
| Sun 2015 | X |  |  |  |  |  |  |  |  |  |  |  |  |  |  |  |  |  |  |
| Tseng 2016 | X |  |  |  |  |  |  |  |  |  |  |  |  |  |  |  |  |  |  |
| Ushida 2020 | X |  |  |  |  |  |  |  |  |  |  |  |  |  |  |  |  |  |  |
| Ushida 2020 | X |  |  |  |  |  |  |  |  |  |  |  |  |  |  |  |  |  |  |
| Wolford 2020 | X |  |  |  |  |  |  |  |  |  |  |  |  |  |  |  | X |  |  |
| Young 2016 | X |  |  |  |  |  |  |  |  |  |  |  |  |  |  |  |  |  |  |
| Carlo 2011 |  |  | X | X |  |  |  |  |  |  |  |  |  |  |  |  |  |  |  |
| Wong 2014 |  |  | X | X |  |  |  |  |  |  |  |  |  |  |  |  |  |  |  |
| Laughon 2019 |  |  | X |  |  |  |  |  |  |  |  |  |  |  |  |  |  |  |  |
| Liu 2012 |  |  |  | X |  |  |  |  |  |  |  |  |  |  |  |  |  |  |  |
| Schaap 2001 |  |  |  | X |  |  |  |  |  |  |  |  |  | X |  |  |  | X |  |
| Doyle 2000 |  |  |  | X |  |  |  |  |  |  |  |  |  |  |  | X |  |  |  |
| Doyle 1989 |  |  |  | X |  |  |  |  |  |  |  |  |  |  |  |  |  |  |  |
| Doyle 1986 |  |  |  | X |  |  |  |  |  |  |  |  |  |  |  |  |  |  |  |
| Crowther 2006 |  |  |  |  |  |  |  | X | X | X |  |  |  |  |  |  |  |  |  |
| Murphy 2008 |  |  |  |  |  |  | X | X | X | X |  |  |  |  |  |  |  |  |  |
| Peltoniemi 2007 |  |  |  |  |  |  | X | X | X | X |  |  |  |  |  |  |  |  |  |
| TEAMS 1999 |  |  |  |  |  |  |  | X | X |  |  |  |  |  |  |  |  |  |  |
| Wapner 2006 |  |  |  |  |  |  | X | X | X | X |  |  |  |  |  |  |  |  |  |
| Vanda 2018 |  |  |  |  |  |  | X |  |  |  |  |  |  |  |  |  |  |  |  |
| Kiran 2007 |  |  |  |  |  |  | X |  |  |  |  |  |  |  |  |  |  |  |  |
| Aghajafari 2002 |  |  |  |  |  |  |  |  | X |  |  |  |  |  |  |  |  |  |  |
| French 1999 |  |  |  |  |  |  |  |  |  |  | X |  |  |  |  |  |  |  |  |
| Crowther 2019 |  |  |  |  |  |  |  |  |  |  |  | X | X |  |  |  |  |  |  |
| Subtil 2003 |  |  |  |  |  |  |  |  |  |  |  | X | X |  |  |  |  |  |  |
| Mitsiakos 2013 |  |  |  |  |  |  |  |  |  |  |  |  |  | X |  |  |  |  |  |
| Kent 2005 |  |  |  |  |  |  |  |  |  |  |  |  |  |  |  |  |  | X |  |
| Leung 2016 |  |  |  |  |  |  |  |  |  |  |  |  |  |  | X |  |  |  |  |
| Kim 2017 |  |  |  |  |  |  |  |  |  |  |  |  |  |  | X |  |  |  |  |
| Waters 2008 |  |  |  |  |  |  |  |  |  |  |  |  |  |  | X |  |  |  |  |
| Chen 2008 |  |  |  |  |  |  |  |  |  |  |  |  |  |  |  | X | X |  |  |
| DeVaries 2008 |  |  |  |  |  |  |  |  |  |  |  |  |  |  |  | X |  |  |  |
| South 2017 |  |  |  |  |  |  |  |  |  |  |  |  |  |  |  | X |  |  |  |
| Nixon 2017 |  |  |  |  |  |  |  |  |  |  |  |  |  |  |  | X |  |  |  |
| Alexander 2016 |  |  |  |  |  |  |  |  |  |  |  |  |  |  |  |  | X |  |  |
| Davis 2013 |  |  |  |  |  |  |  |  |  |  |  |  |  |  |  |  | X |  |  |
| Erni 2012 |  |  |  |  |  |  |  |  |  |  |  |  |  |  |  |  | X |  |  |
| Ghosn 2021 |  |  |  |  |  |  |  |  |  |  |  |  |  |  |  |  | X |  |  |
| Grant 2021 |  |  |  |  |  |  |  |  |  |  |  |  |  |  |  |  | X |  |  |
| Ilg 2018 |  |  |  |  |  |  |  |  |  |  |  |  |  |  |  |  | X |  |  |
| Kang 2021 |  |  |  |  |  |  |  |  |  |  |  |  |  |  |  |  | X |  |  |
| Khalife 2013 |  |  |  |  |  |  |  |  |  |  |  |  |  |  |  |  | X |  |  |
| Ligges 2010 |  |  |  |  |  |  |  |  |  |  |  |  |  |  |  |  | X |  |  |
| Paules 2005 |  |  |  |  |  |  |  |  |  |  |  |  |  |  |  |  | X |  |  |
| Raikkonen 2022 |  |  |  |  |  |  |  |  |  |  |  |  |  |  |  |  |  |  | X |
| Osteen 2022 |  |  |  |  |  |  |  |  |  |  |  |  |  |  |  |  |  |  | X |
