# Supplementary material for: Long-term outcomes of antenatal corticosteroids for preterm birth: An overview of systematic reviews
Source: PLOS Glob Public Health. 2025 May 7;5(5):e0004575. doi: 10.1371/journal.pgph.0004575 (PMC12057917; doi:10.1371/journal.pgph.0004575)
Supplement: Attachment — Submitted filename: PLOS ACS long term.docx [file pgph.0004575.s008.docx]

Very rigorously done and well written! Congratulations.

Table 1 :

-Sotiriadis : « the effects », instead of « to effects », or “to assess effects” ; sometimes it is written “to assess the effects” and sometimes “to assess effects”, could you make it uniform?

Outcomes in the systematic reviews are often assessed within a subgroup of the population that received antenatal corticosteroids, be it only in the premature born children or in the (late pre)term born children. Looking at outcomes of children within such subgroups, poses a major risk of selection bias. This needs to be mentioned in the discussion.
