# Supplementary material for: Long-term outcomes of antenatal corticosteroids for preterm birth: An overview of systematic reviews
Source: PLOS Glob Public Health. 2025 May 7;5(5):e0004575. doi: 10.1371/journal.pgph.0004575 (PMC12057917; doi:10.1371/journal.pgph.0004575)
Supplement: Attachment — Submitted filename: Response letter.docx [file pgph.0004575.s011.docx]

The Editor

PLOS Global Public Health

Dear editor,

Thank you for taking the time to review our manuscript, and to provide detailed comments and suggestions. We have responded to the reviewers’ comments in the point-by-point response below.

** All line numbers in the response section refer to line numbers in the marked copy of manuscript when viewing in “All Markup” mode.

**Reviewer 1**

Dear Authors
Your review is very interesting But I have some recommends

1. In Your review have some contradictory data according to multiple course of ACST and its relation to neurodevelopmental in neonate, childhood as well as in the adults.

**Response:** Thank you for highlighting this important point about the data on multiple ACST courses. Our review findings suggest that, compared to a single course of ACS, multiple courses of ACS might be associated with adverse neurodevelopmental outcomes in children born at term, while there were no long-term benefits or harms observed in preterm-born children. However, overall findings were based on limited data from a small number of studies with modest sample sizes, which may be underpowered to detect clinically meaningful differences. Thus, caution is warranted while interpreting these findings. The discussion section of our manuscript already addresses these differences and acknowledges the limitations of the available evidence.

2. Does Authors recommend single or multiple courses of ACST in preventing long time sequels.

**Response:** We are not able to make a definitive recommendation about single or multiple courses of ACST based solely on long-term outcomes due to the methodological limitations within the studies, including insufficient evidence from efficacy trials. This underscores the need for further high-quality research to generate a robust evidence base on long term outcomes. Meanwhile, we recommend that clinicians carefully evaluate the likelihood of birth after 34 weeks' gestation before administering ACS to optimize outcomes and minimize potential adverse effects. However, it seems likely that ACS exposure in a baby born at term is counter-productive.

3. Authors did not emphases long-time sequels and differences between single maternal beta and dexamethasone therapy relation to cardiovascular and metabolism in adults.

**Response:** Thank you for your comment. We have now incorporated this in the discussion.

Lines- 416 – 426

Understanding the long-term effects of ACS during adulthood is also critical, given the growing recognition of early-life events on the development of chronic diseases and the potential role of glucocorticoid exposure in these relationships (48). Preclinical animal studies have demonstrated that ACS exposure is associated with increased risks of cardiovascular, metabolic and other diseases in adult offspring (49-52). However, human studies examining long-term cardiometabolic outcomes in adults exposed to ACS remain limited, and their findings are inconclusive, with most evidence derived from observational studies. Some studies found no association between ACS exposure and cardiometabolic disease risk in later life (53, 54), while others reported elevated blood pressure, increased aortic stiffness, and dysregulated glucose metabolism (55-57). Hence, longitudinal studies of trial cohorts are needed to definitively evaluate the effects of ACS throughout the life-course.

**Reviewer 2:**

1. Very rigorously done and well written! Congratulations.

**Response:** Many thanks for your positive comments.

2. Table 1: -Sotiriadis: « the effects », instead of « to effects », or “to assess effects”; sometimes it is written “to assess the effects” and sometimes “to assess effects”, could you make it uniform?

**Response:** Thank you for pointing this out. We have now corrected it in Table 1 (Page 10).

3. Outcomes in the systematic reviews are often assessed within a subgroup of the population that received antenatal corticosteroids, be it only in the premature born children or in the (late pre)term born children. Looking at outcomes of children within such subgroups, poses a major risk of selection bias. This needs to be mentioned in the discussion.

**Response:** Thank you for this suggestion. We have incorporated this in the discussion.

Lines 357 – 360

Additionally, assessing outcomes only in specific subgroups (e.g., gestational age) may introduce selection bias, potentially masking true treatment effects. These analyses often inadequately capture the inherent variability present in the broader study population.

**Additional responses in relation to Journal Requirements**

1. As required by our policy on Data Availability, please ensure your manuscript or supplementary information includes the following:

A numbered table of all studies identified in the literature search, including those that were excluded from the analyses.

For every excluded study, the table should list the reason(s) for exclusion.

**Response:** The list of excluded studies with reason(s) for exclusion is available in **S3 appendix.**

2. If any of the included studies are unpublished, include a link (URL) to the primary source or detailed information about how the content can be accessed.

**Response:** Not applicable

3. A table of all data extracted from the primary research sources for the systematic review and/or meta-analysis. The table must include the following information for each study:

Name of data extractors and date of data extraction

Confirmation that the study was eligible to be included in the review.

All data extracted from each study for the reported systematic review and/or meta-analysis that would be needed to replicate your analyses.

**Response:** We have now uploaded the data extraction file as supporting information in **S2 appendix**.

4. If data or supporting information were obtained from another source (e.g. correspondence with the author of the original research article), please provide the source of data and dates on which the data/information were obtained by your research group.

**Response:** Not applicable

5. If applicable for your analysis, a table showing the completed risk of bias and quality/certainty assessments for each study or outcome.  Please ensure this is provided for each domain or parameter assessed. For example, if you used the Cochrane risk-of-bias tool for randomized trials, provide answers to each of the signalling questions for each study. If you used GRADE to assess certainty of evidence, provide judgements about each of the quality of evidence factor. This should be provided for each outcome.

**Response:** Quality assessment reports are available in **S1 Table.**

6. An explanation of how missing data were handled.

**Response:** Not applicable
