# Supplementary material for: Long-term outcomes of antenatal corticosteroids for preterm birth: An overview of systematic reviews
Source: PLOS Glob Public Health. 2025 May 7;5(5):e0004575. doi: 10.1371/journal.pgph.0004575 (PMC12057917; doi:10.1371/journal.pgph.0004575)
Supplement: Attachment — Submitted filename: PLOS ACS long term_review.docx [file pgph.0004575.s010.docx]

Could you please change the sentence you added by this sentence (or an alternative of your choice which covers the topic)?

Additionally, assessing outcomes only in specific subgroups (e.g., gestational age) within one treatment arm may introduce bias. Within these subgroups, the comparison is not between no treatment or placebo and treatment, but between the subgroups (all under treatment or no treatment/placebo), which cannot be subject of randomization.
