# Supplementary material for: Long-term outcomes of antenatal corticosteroids for preterm birth: An overview of systematic reviews
Source: PLOS Glob Public Health. 2025 May 7;5(5):e0004575. doi: 10.1371/journal.pgph.0004575 (PMC12057917; doi:10.1371/journal.pgph.0004575)
Supplement: Attachment — Submitted filename: Response_letter_auresp_2.docx [file pgph.0004575.s012.docx]

The Editor

PLOS Global Public Health

Dear editor,

Thank you for taking the time to review our manuscript. We have responded to the reviewer’s comment in the point-by-point response below.

** All line numbers in the response section refer to line numbers in the marked copy of manuscript when viewing in “All Markup” mode.

Could you please change the sentence you added by this sentence (or an alternative of your choice which covers the topic)?

Additionally, assessing outcomes only in specific subgroups (e.g., gestational age) within one treatment arm may introduce bias. Within these subgroups, the comparison is not between no treatment or placebo and treatment, but between the subgroups (all under treatment or no treatment/placebo), which cannot be subject of randomization.

**Response:** Thank you for your suggestion. We have now incorporated this in the discussion. We would like to point out that our original statement regarding potential for selection bias pertained to data from observational studies, rather than from randomized trials. We have therefore opted to remove the reference to randomization.

***Lines: 357 – 361***

Additionally, considering outcomes only for a selected subgroup (e.g., a gestational age subgroup, such as preterm infants only) may introduce selection bias, potentially masking true treatment effects. When comparing different subgroups, it is essential to exercise caution by analyzing both treatment and control groups, rather than examining subgroups within only one group.
